# Enhancing breadth and durability of humoral immune responses in non-human primates with an adjuvanted group 1 influenza hemagglutinin stem antigen

**DOI:** 10.1038/s41541-023-00772-1

**Published:** 2023-11-11

**Authors:** Maarten Swart, Harmjan Kuipers, Fin Milder, Mandy Jongeneelen, Tina Ritschel, Jeroen Tolboom, Leacky Muchene, Joan van der Lubbe, Ana Izquierdo Gil, Daniel Veldman, Jeroen Huizingh, Johan Verspuij, Sonja Schmit-Tillemans, Sven Blokland, Martijn de Man, Ramon Roozendaal, Christopher B. Fox, Hanneke Schuitemaker, Martinus Capelle, Johannes P. M. Langedijk, Roland Zahn, Boerries Brandenburg

**Affiliations:** 1 Janssen Vaccines & Prevention, Leiden, The Netherlands; 2 Access to Advanced Health Institute, Seattle, WA USA

**Keywords:** Adjuvants, Protein vaccines

## Abstract

Seasonal influenza vaccines must be updated annually and suboptimally protect against strains mismatched to the selected vaccine strains. We previously developed a subunit vaccine antigen consisting of a stabilized trimeric influenza A group 1 hemagglutinin (H1) stem protein that elicits broadly neutralizing antibodies. Here, we further optimized the stability and manufacturability of the H1 stem antigen (H1 stem v2, also known as INFLUENZA G1 mHA) and characterized its formulation and potency with different adjuvants in vitro and in animal models. The recombinant H1 stem antigen (50 µg) was administered to influenza-naïve non-human primates either with aluminum hydroxide [Al(OH)_3_] + NaCl, AS01_B_, or SLA-LSQ formulations at week 0, 8 and 34. These SLA-LSQ formulations comprised of varying ratios of the synthetic TLR4 agonist ‘second generation synthetic lipid adjuvant’ (SLA) with liposomal QS-21 (LSQ). A vaccine formulation with aluminum hydroxide or SLA-LSQ (starting at a 10:25 µg ratio) induced HA-specific antibodies and breadth of neutralization against a panel of influenza A group 1 pseudoviruses, comparable with vaccine formulated with AS01_B_, four weeks after the second immunization. A formulation with SLA-LSQ in a 5:2 μg ratio contained larger fused or aggregated liposomes and induced significantly lower humoral responses. Broadly HA stem-binding antibodies were detectable for the entire period after the second vaccine dose up to week 34, after which they were boosted by a third vaccine dose. These findings inform about potential adjuvant formulations in clinical trials with an H1 stem-based vaccine candidate.

## Introduction

While influenza transmission was reduced during the COVID-19 pandemic, the burden of influenza-related disease increased again after relaxation of public health measures combined with reduced population immunity^1^. Influenza is estimated to cause 3–5 million cases of severe illness and is responsible for 290,000 to 650,000 deaths annually worldwide (pre-COVID-19 pandemic)^2^. Vaccination is considered to be one of the most cost-effective health interventions to prevent influenza^3^. Current influenza vaccines may need to be updated annually as influenza viruses continue to evolve by acquiring mutations that enable it to evade protection by previous infections or vaccination. These seasonal influenza vaccines predominantly protect against strains closely related to the strains the vaccine is based on, but provide little protection against other influenza strains or subtypes^4,5^. The identification of broadly protective neutralizing antibodies fueled the development of a class of universal or broadly protective influenza vaccines, targeting conserved epitopes in the haemagglutinin (HA) protein^6^. As these broadly protective antibodies are predominantly directed to the highly conserved stem region of HA, we developed a subunit protein vaccine consisting of a stabilized trimeric influenza A group 1 HA (H1) stem without the immune-dominant HA head domain^7^. Vaccination with this H1 stem protein protected against challenge with heterosubtypic influenza A group 1 challenge in mice and in non-human primates (NHP) when administered with aluminum salts^7–9^.

Adjuvants are thought to contribute to a better quality of the HA stem-specific immune response by increased recruitment of (memory) B cells and follicular T helper cells^10,11^. We therefore set out to further improve the immunogenicity of H1 stem by screening and optimizing various adjuvant formulations. Although aluminum salts induce humoral responses and are among the most widely used adjuvants, alternative adjuvants have been developed that induce superior antibody levels compared to aluminum salts^12,13^. AS01_B_ was developed to elicit strong cellular and humoral responses^12,14^ and is included in the herpes zoster (*Shingrix*) vaccine^15,16^. AS01_B_ consists of a saponin fraction (QS-21)^17^ and the toll-like receptor 4 agonist (TLR4) 3-O-desacyl-4′- monophosphoryl lipid A (MPL^®^) that is purified from *Salmonella minnesota*, in a liposomal formulation^18^. MPL^®^ is a heterogeneous mixture of congeneric lipid A with 4′-phophoryl groups and varying numbers of acyl chains with hexa- and penta-acylated species reported to be TLR4 agonists, while tetra- and tri-acylated species are human TLR4 antagonists^19^. As MPL^®^ is also believed to be responsible for the local injection site pain observed in human vaccinees^20^, careful titration of the adjuvant components is required to achieve similar levels of immunogenicity compared with AS01_B_, while potentially reducing the reactogenicity following vaccination in humans. Further development of adjuvant components has led to a second-generation synthetic lipid adjuvant (SLA) TLR4 agonist, which is a hexa-acylated structure, to further improve the binding affinity to human TLR4 and improve its adjuvanting properties^21,22^. In this study, we evaluated the immunogenicity of H1 stem formulated with different doses of SLA and QS-21 in a liposomal formulation (SLA-LSQ)^23^ in NHP, in comparison with AS01_B_.

## Results

### Design and in vitro characterization of H1 stem antigens

**Fig. 1:**
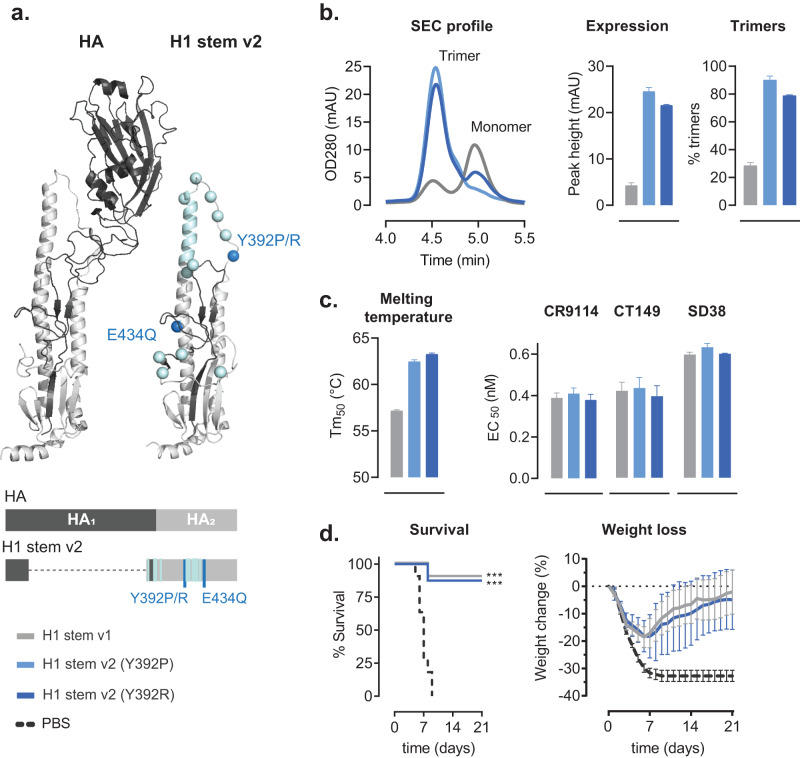
Characterization of H1 stem antigens. **a** Structure (top) and a schematic representation (below) of monomeric HA (PDB ID 4M4Y) and a model of monomeric H1 stem v2. H1 stem v1 design mutations that are also included in H1 stem v2 are indicated in pale cyan (point mutations and the GCN4 trimerization motif. The two additional manufacturing-improving mutations in H1 stem v2 are highlighted in blue. **b** SEC analysis of Expi293F cell culture supernatant expressing H1 stem v1 and H1 stem v2 (Y392P or R). Profiles represent the mean of three independent transfections and peaks corresponding to monomeric and trimeric species are indicated (left panel). Expression (middle panel) and percentage trimer (right panel) shown as bar plots. Data are expressed as mean ± standard deviation. **c** In vitro characterization of trimeric H1 stem protein purified from ExpiCHO cell cultures. Temperature stability as determined by DSF (*n* = 3) and expressed as mean Tm_50_ values (left panel). Binding of a panel of antibodies (*n* = 3) as measured by ELISA are expressed as EC_50_ values for H1 stem v1 and H1 stem v2 (right panel). Data are expressed as mean ± standard deviation. **d** Survival and weight loss after lethal H1N1 A/Brisbane/59/2007 challenge. Mice were intramuscularly immunized with 30 μg H1 stem v1 as described by Impagliazzo et al.^7^ (*n* = 10), H1 stem v2 (Y392R, *n* = 8), both adjuvanted with 50 μg Al(OH)_3_ on day −49 and −28. Animals vaccinated with PBS were included as a control (*n* = 11). Post-challenge survival until day 21 after challenge with 12.5xLD_50_ of H1N1 A/Brisbane/59/2007 at day 0 is shown. The median weight loss per groups is shown with the inter-quartile range. Statistical comparisons were performed using a 2-sided Fisher’s exact test. Significant differences in survival compared with the PBS group are indicated by asterisks: ***P < 0.001.

To increase protein stability and manufacturability, two additional point mutations were introduced to the original H1 stem design^7^ (referred to as H1 stem v1) to obtain a H1 stem v2 design. An important region of instability in influenza A HA is the second pH-sensitive switch at the bend of the central helix D to helix E^24^. The negatively charged Glu434 at this interface was substituted with the neutral Gln, stabilizing this switch region in the trimer interface by neutralizing the repulsion and optimizing the interaction with the 30-loop. Previously, residues at several positions in the B-loop were successfully optimized to generate a stable soluble stem-based immunogen^7^. Position 392 in the B-loop of the current construct was further explored for optimization by substitutions to the charged Arg or to a Pro which is known to potentially stabilize the hinge region of class I fusion proteins^25–27^ (Fig. 1a). Supernatants of cells transfected with plasmids encoding H1 stem v1 and the improved H1 stem v2 design were tested for expression and trimer content (Fig. 1b). The two mutations increased the expression yields of trimeric H1 stem v2 by ∼5-5.7-fold and the percentage trimers by ∼2.7-3.2-fold, compared with H1 stem v1. Trimeric H1 stem v1 and H1 stem v2 were purified for further testing. The improved purified trimeric H1 stem v2 showed an increase in melting temperature (Tm_*50*_) up to 6.1 °C as determined by differential scanning fluorimetry (DSF), whereas antigenicity was maintained as confirmed by EC_50_ binding values (ELISA) in the lower nanomolar range as shown for monoclonal (CR9114^28^ and CT149^29^) and multidomain (SD38-Fc^30^) antibodies (Fig. 1c). H1 stem v2 Y392R was selected as it had the highest melting temperature, while antigenicity was comparable between H1 stem v2 Y392P and Y392R. Next, we evaluated the protective efficacy of H1 stem v1 and -v2 (Y392R) designs in a lethal H1N1 A/Brisbane/59/2007 influenza challenge model. We observed comparable, significant protection by both H1 stem v1 and v2 compared to PBS, indicating that increased protein stability and manufacturability did not affect immunogenicity (Fig. 1d).

### Optimization of the aluminum-based adjuvants

We have previously shown that the H1 stem antigen is immunogenic in mice and NHP when administered with the salt aluminum hydroxide [Al(OH)_3_] diluted in PBS^7–9^. In addition to Al(OH)_3_, aluminum phosphate (AlPO_4_) is commonly used and has different biological and physicochemical properties^31^. Antigen adsorption to the adjuvant is thought to be one of the factors driving immunogenicity^32^. We found that less than 20% H1 stem v2 was bound to AlPO_4_, while antigen adsorption was highest to Al(OH)_3_ in a buffer containing 2% sucrose/ 10 mM Tris ( >90% bound) and 70-80% bound for Al(OH)_3_ diluted in PBS (Table 1). Art et al*.* reported that increasing the salt concentration increases Al(OH)_3_ particle aggregation^32^, which is thought to influence immunogenicity. Therefore, we also formulated H1 stem v2 in Al(OH)_3_ + 140 mM NaCl.

**Table 1:** Characteristics of different aluminum salt-based adjuvant formulations.

Adjuvant	Formulation	pH	Bound protein^a^
AlPO_4_	2% sucrose, 10 mM Tris	8	<20%
Al(OH)_3_	2% sucrose, 10 mM Tris	8	>90%
Al(OH)_3_ + PBS	1 mM KH_2_PO_4_, 3 mM Na_2_HPO_4_, 155 mM NaCl	7.4	70 – 80%
Al(OH)_3_ + NaCl	2% sucrose, 5 mM Tris, 140 mM NaCl	8	>90%

^a^The amount of adsorbed H1 stem v2 protein was determined indirectly by measuring the residual protein in the supernatant after centrifugation of the protein-adjuvant mixture.

**Fig. 2:**
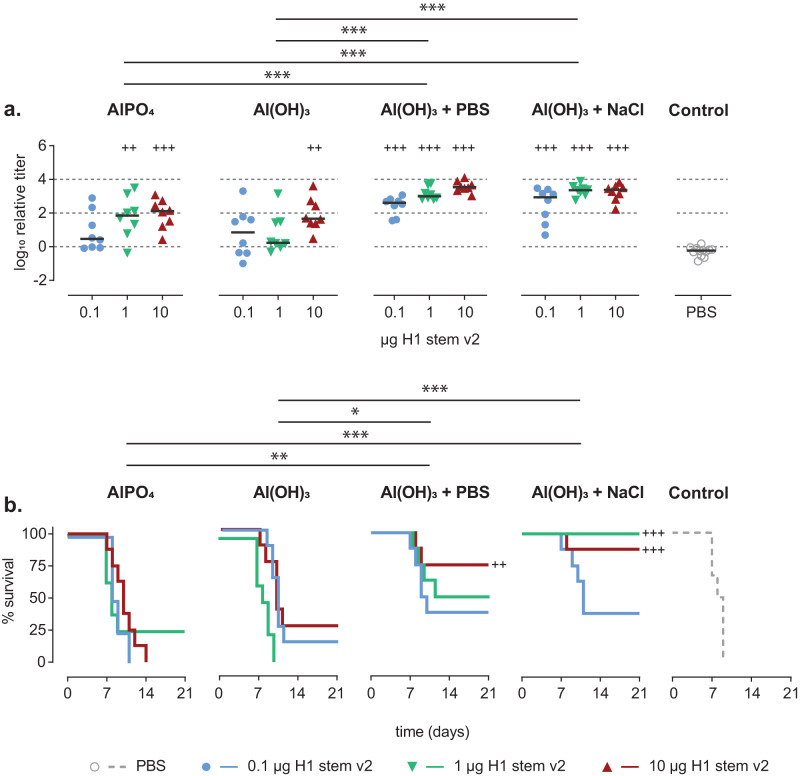
Formulation of Al(OH)_3_ + NaCl enhances the immunogenicity and efficacy of H1 stem v2 in mice. Mice were immunized with 0.1, 1 or 10 μg H1 stem v2 adjuvanted with 50 μg 2% AlPO_4_, Al(OH)_3_, Al(OH)_3_ + PBS or Al(OH)_3_ + NaCl twice on day −49 and −28 (*n* = 8 per dose). Animals vaccinated with PBS (*n* = 8) were included as a control. **a** HA stem specific binding antibody levels were measured at day −1 using a H1N1 A/California/07/09 full-length HA IgG-binding ELISA. Individual binding titers per animal are shown. Black horizontal bars indicate the group medians. **b** Survival proportions are shown post-challenge with 12.5xLD50 of H5N1 A/Hong Kong/156/1997 until day 21. Comparisons with the control group or across dose comparisons were performed using an ANOVA (**a**) or a 2-sided Fisher’s exact test (**b**) with a 4-fold Bonferroni-correction. Statistical differences compared with the control group are indicated with plus symbols: ++*P* < 0.01, +++*P* < 0.001 and statistical differences across dose are indicated by asterisks: *P < 0.05, **P < 0.01, ***P < 0.001.

To evaluate which aluminum-based adjuvant maximized the immunogenicity of H1 stem v2, mice were vaccinated twice with three dose levels (0.1, 1 or 10 μg) of H1 stem v2 adjuvanted with 50 μg 2% AlPO_4_, Al(OH)_3_, Al(OH)_3_ + PBS or Al(OH)_3_ + NaCl. HA stem-binding antibody titers were highest across dose in mice immunized with H1 stem v2 adjuvanted with either Al(OH)_3_ + PBS or Al(OH)_3_ + NaCl (Fig. 2a), compared with AlPO_4_ and Al(OH)_3_. Subsequently, the mice received a lethal H5N1 A/Hong Kong/156/1997 influenza challenge to assess the breadth of protection, as H5N1 is a different subtype compared to the H1N1-derived H1 stem v2. In addition, H5N1 is of interest as it has pandemic potential in humans. While we did not observe protection in the AlPO_4_- and Al(OH)_3_-adjuvanted groups against a lethal challenge compared with the control-immunized group, mice immunized with an H1 stem v2 dose from 10 μg adjuvanted with Al(OH)_3_ + PBS and as low as 1 μg adjuvanted with Al(OH)_3_ + NaCl were significantly protected (Fig. 2b).

### In vitro characterization of SLA-LSQ formulations

It has recently been shown that levels of HA stem antibodies can be increased with the next generation adjuvants AS01_B_ and AS03^33^. Therefore, we also aimed to evaluate the immunogenicity of H1 stem v2 adjuvanted with AS01_B_ or SLA-LSQ, which consists of the synthetic TLR4 agonist SLA and a saponin fraction (QS-21) in a liposomal formulation. MPL^®^, which is part of AS01_B_, is a mixture of TLR4 agonists and antagonists^19^ and believed to be responsible for the local injection site pain observed in human vaccinees^20^. Therefore, careful titration of the adjuvant dose is required to achieve similar levels of immunogenicity compared with AS01_B_, while potentially reducing the reactogenicity following vaccination in humans.

**Fig. 3:**
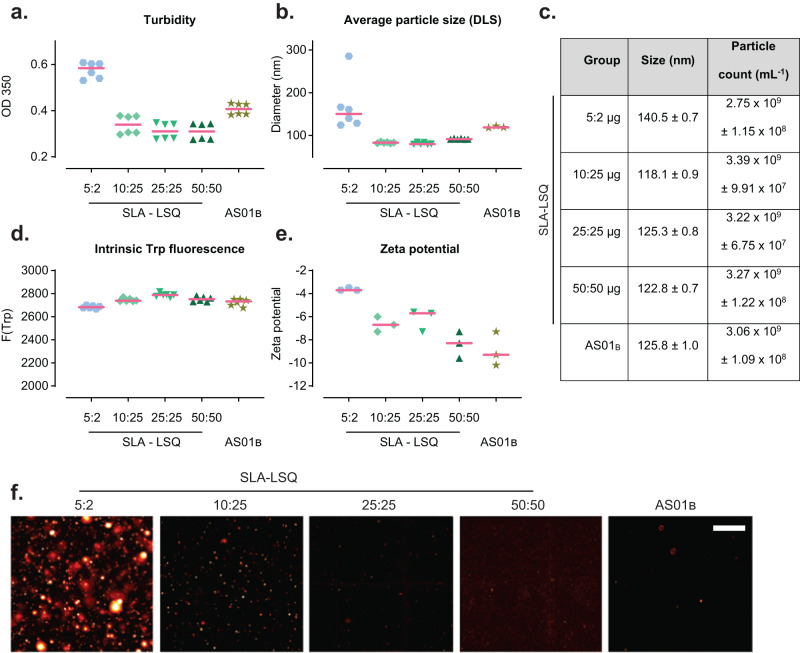
In vitro characterization of H1 stem v2 formulated with SLA-LSQ and AS01_B_. H1 stem v2 was formulated with SLA-LSQ containing SLA and QS-21 in a ratio of 5:2 μg, 10:25 μg, 25:25 μg or 50:50 μg or AS01_B_ containing 50:50 μg MPL^®^ and QS-21. **a** Absorption at 350 nm indicates the turbidity. **b** Average particle size was evaluated using dynamic light scattering (DLS) and **c** nanoparticle tracking analysis (NTA) in nm. The particle count was also evaluated using NTA in count/ mL. **d** Group means are shown ±standard deviation. The peak intrinsic tryptophan fluorescence was measured at 340 nm after excitation at 280 nm. **e** The zeta potential was measured using the zetasizer nano ZS. **f** The larger micrometer-size globular and hydrophobic structures were visualized by Nile Red staining. Red horizontal bars in (**a**), (**b**), (**d**) and (**e**) indicate the group medians. Scale bar indicates 100 μm.

First, various analytical methods were applied to characterize the AS01_B_ and SLA-LSQ vaccine formulations in vitro before and after mixing with H1 stem v2. We observed a higher absorbance at 350 nm in the 5:2 μg SLA-LSQ formulation (Fig. 3a), indicating an increase in turbidity (cloudiness). The average particle size as quantified using dynamic light scattering (Fig. 3b) and nanoparticle tracking analyses showed an increase in particle size (Fig. 3c). In addition, nanoparticle tracking revealed a reduced number of larger particles. All formulations had similar intrinsic tryptophan fluorescence (Fig. 3d), indicating that there is no interaction of the protein with the lipid bilayer of the liposomes or larger formed lipid-like structures. The H1 stem v2 protein therefore does not contribute to additional fusion or aggregation of the SLA-LSQ formulations. However, we observed a higher zeta potential in the 5:2 μg SLA-LSQ formulation (Fig. 3e) that may be responsible for the fusion/ aggregation of liposomes as there are less repulsive charges present. The presence of globular particles in the micrometer range in the 5:2 μg SLA-LSQ formulation were visualized by microscopy after Nile Red staining (Fig. 3f). When analyzing the formulation characteristics of adjuvants without antigen, 5:2 μg SLA-LSQ was also more turbid and contained larger particles before mixing with H1 stem v2, suggesting that these aggregates were not introduced upon mixing with H1 stem v2 (Supplemental Fig. 1a, b). Staining with Nile Red confirmed these findings as the 5:2 μg SLA-LSQ group showed reduced bulk fluorescence due to a reduced total surface area and clearly visible aggregates when visualized with microscopy (Supplemental Fig. 1c, d).

### Humoral responses after vaccination of cynomolgus monkeys with adjuvanted H1 stem HA

MPL^®^ contains tetra- and tri-acylated congeneric lipid A species that are TLR4 antagonists in humans^19^, but are agonists in mice^19^. Therefore, we evaluated the reactogenicity and immunogenicity of H1 stem v2 adjuvanted with SLA-LSQ in cynomolgus monkeys, as they have a similar TLR receptor distribution compared to humans^34^. Monkeys received a dose of H1 stem v2 on week 0 and week 8. Subsequently the animals received a dose at week 24 to evaluate amnestic responses. AS01_B_ (containing 50:50 μg MPL^®^ and QS-21) and Al(OH)_3_ + NaCl were included as reference adjuvants. Different doses of SLA-LSQ with a ratio of 5:2 μg, 10:25 μg, 25:25 μg or 50:50 μg SLA and QS-21 were included to evaluate if comparable immunogenicity to AS01_B_ can be achieved at lower adjuvant doses. Local injection-site reactions were monitored using the Draize scoring system post immunization to assess skin reactogenicity. However, we did not observe edema and erythema at the injection site up to three days after the first two doses. Only minimal erythema was detected after the third dose in one animal that received H1 stem v2 adjuvanted with 25:25 μg SLA-LSQ and two animals that received Al(OH)_3_ + NaCl as an adjuvant.

**Fig. 4:**
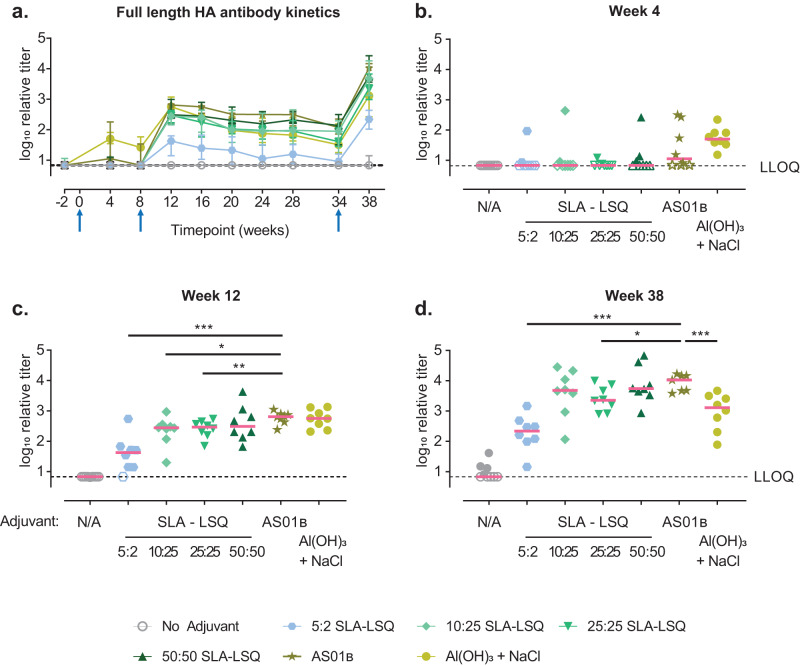
Influenza haemagglutinin stem-specific binding antibodies after vaccination of NHPs with adjuvanted H1 stem protein. Influenza-seronegative cynomolgus macaques received either unadjuvanted H1 stem v2 or H1 stem v2 adjuvanted with SLA-LSQ containing SLA and QS-21 in a ratio of 5:2 μg, 10:25 μg, 25:25 μg or 50:50 μg, H1 stem v2 adjuvanted with AS01_B_ containing 50:50 μg MPL^®^ and QS-21 or Al(OH)_3_ + NaCl (*N* = 8-9) in a 3-dose immunization regimen on week 0, 8 and 34. **a** HA stem-specific binding antibody levels were measured at week −2, 4, 8, 12, 16, 20, 24, 28, 34 and 38 using a H1N1 A/California/07/09 full-length HA IgG-binding ELISA. Median binding titers per group are shown and error bars denote the interquartile range (IQR). Individual binding titers per animal are shown for week (**b**) 4, (**c**) 12 and (**d**) 38. Red horizontal bars indicate the group medians and the dotted line indicates the lower limit of quantification (LLOQ). Open symbols indicate the response is at or below the LLOQ. Comparisons between the SLA-LSQ- and Al(OH)_3_ + NaCl-vaccine groups with the AS01_B_ group were performed by a Wilcoxon rank sum test. Statistical differences are indicated by asterisks: *P < 0.05, **P < 0.01, ***P < 0.001. Additional statistical comparisons are shown in Supplemental Tables 1–3.

HA stem-binding antibodies were measured using a H1N1 A/California/07/2009 full-length HA IgG-binding ELISA (Fig. 4a). Although no statistical significant differences in mean binding-antibody titers were detected between the SLA-LSQ and Al(OH)_3_ + NaCl groups with AS01_B_ 4 weeks post dose 1 (Fig. 4b), the number of animals with HA stem-binding antibodies above the lower limit of quantification (LLOQ) appeared to be higher in the Al(OH)_3_ + NaCl adjuvanted group at this time point. Four weeks post dose 2 (study week 12), HA stem-binding antibodies were detected in all adjuvanted groups. HA IgG antibody-binding titers were not different between the Al(OH)_3_, the 50:50 μg SLA-LSQ and the AS01_B_ group (Fig. 4c). However, binding antibody titers were lower in the groups adjuvanted with 5:2, 10:25 and 25:25 μg SLA-LSQ. The antibody titers gradually decreased over time after week 12, until the animals received a third dose at week 34 (Fig. 4a). At week 38, antibody titers were lower in the Al(OH)_3_ + NaCl-adjuvanted group, compared with the AS01_B_-adjuvanted group. However, 10:25 and 50:50 μg SLA-LSQ induced comparable binding-antibody titers with AS01_B_, while titers were lower in the 5:2 and the 25:25 μg SLA-LSQ groups (Fig. 4d).

Comparable results were obtained with a H1N1 A/California/07/2009 HA stem IgG-binding ELISA (Supplemental Fig. 2a–c). Moreover, we measured HA stem-binding titers using this ELISA in a cohort of healthy human volunteers that received the seasonal trivalent influenza vaccine Inflexal V that included the influenza strains H1N1 A/California/07/2009, H3N2 A/Victoria/210/2009 and B/Brisbane/60/2008^35^. The adult population generally has pre-existing HA stem binding antibodies from previous infections or vaccinations. Seasonal influenza vaccination led to an approximately 2-fold increase in HA stem binding antibodies titers in humans, but antibody titers were relatively low (Supplemental Fig. 2d). By contrast, we observed both higher HA stem binding antibody titer increases as well as higher absolute antibody levels after 3 doses of adjuvanted H1 stem v2 in NHPs compared with seasonal influenza vaccination in humans (Supplemental Fig. 2c). This suggests that vaccination with H1 stem v2 has the potential to increase HA stem antibody levels in humans as well.

**Fig. 5:**
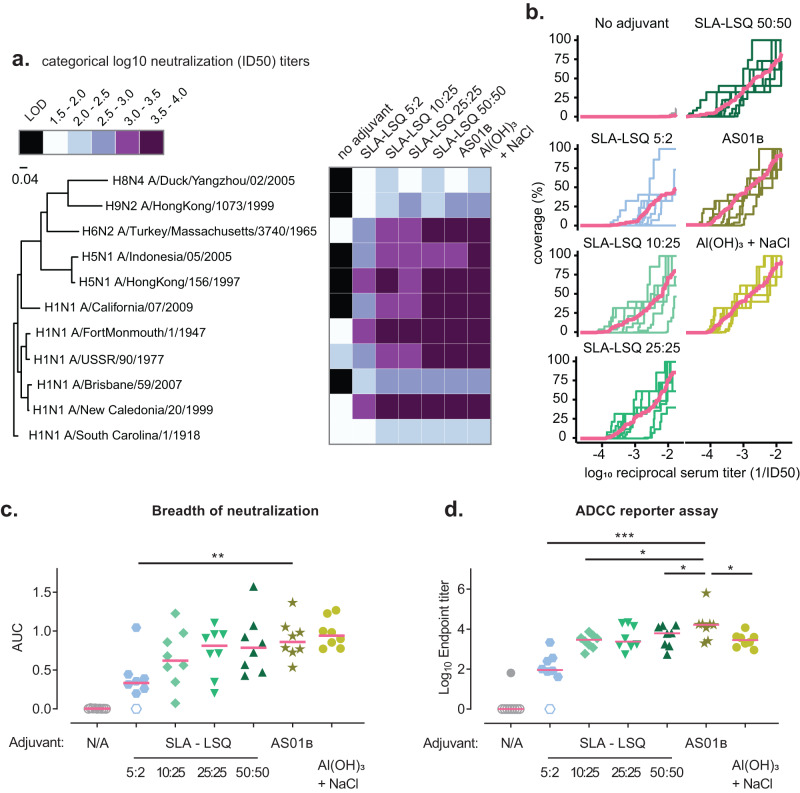
Neutralizing breadth induced by vaccination of NHPs with adjuvanted H1 stem protein. Influenza-seronegative cynomolgus macaques received either unadjuvanted H1 stem v2 or H1 stem v2 adjuvanted with SLA-LSQ containing SLA and QS-21 in a ratio of 5:2 μg, 10:25 μg, 25:25 μg or 50:50 μg, H1 stem v2 adjuvanted with AS01_B_ containing 50:50 μg MPL^®^ and QS-21 or Al(OH)_3_ + NaCl (*N* = 8–9) on week 0 and 8. **a** Neutralizing antibody titers were measured using a ppVNA against a panel of influenza A group 1 virus strains at week 12 (4 weeks post dose 2). The median neutralizing titers per group are visualized in a heatmap using binned neutralization titers. **b** Breadth-potency curves of individual animals per group. Breadth denotes the neutralization coverage of the panel of ppVNA influenza strains as shown in (**a**). Red line represents the mean curve per group. **c** AUC of each breadth-potency curve as shown in (**b**). **d** ADCC was quantified using a H1N1 A/California/07/09 HA-specific hFcγRIIIa reporter assay at week 12. Endpoint titers are shown for individual animals. Red horizontal bars indicate the group medians and open symbols indicate the AUC for the breadth of neutralization is 0 or that ADCC was not detected. Comparisons between the SLA-LSQ- and Al(OH)_3_ + NaCl-vaccine groups with the AS01_B_ group were performed by a Wilcoxon rank sum test. Statistical differences are indicated by asterisks: *P < 0.05, **P < 0.01, ***P < 0.001. Additional statistical comparisons are shown in Supplemental Tables 4 and 5.

Influenza virus neutralization breadth was also evaluated at week 12 using a pseudovirus entry inhibition assay (pseudo-particle Virus Neutralization Assay, ppVNA), using pseudotyped retroviruses expressing a large panel of HA and NA Influenza A group 1 strains. Broad neutralization was observed, with neutralizing titers detected against most strains (Fig. 5a; see Supplemental Fig. 3 for individual results). We detected significantly lower neutralizing titers against all variants tested in animals that had received H1 stem v2 adjuvanted with 5:2 μg SLA-LSQ, compared with AS01_B_. The area under the curve (AUC) of breadth-potency plots was calculated to quantify the breadth of neutralization, which also showed a significantly lower AUC in the 5:2 μg SLA-LSQ group (Fig. 5b, c). No significant differences were found between the SLA-LSQ groups starting at 10:25 μg and the Al(OH)_3_ + NaCl-group, with AS01_B_.

Non-neutralizing HA stem antibodies have also been shown to contribute to protection by ADCC of infected cells upon the interaction with FcγRIIIa on natural killer (NK) cells^36,37^. A reporter assay was used in which cells express luciferase upon binding of FcγRIIIa with the Fc of antigen-bound antibodies that correlates with functional ADCC assays^38^. 4 weeks post dose 2 (week 12), FcγRIIIa signaling was comparable in the groups that received 25:25 μg SLA-LSQ and AS01_B_. However, FcγRIIIa signaling was significantly lower in the 5:2, 10:25 and 50:50 μg SLA-LSQ groups (Fig. 5d). In addition, HA stem-binding antibodies partially correlated with the AUC for neutralization and ADCC titers (Supplemental Fig. 4).

### Cellular responses after vaccination of cynomolgus monkeys with adjuvanted H1 stem protein

**Fig. 6:**
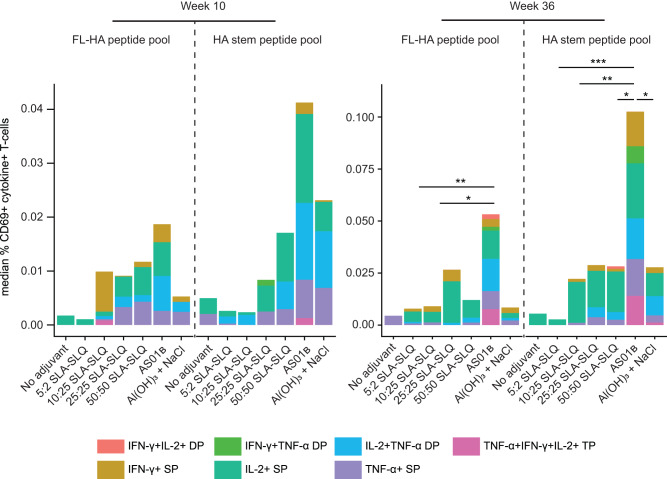
CD4 responses induced by vaccination of NHPs with adjuvanted H1 stem protein. Influenza-seronegative cynomolgus macaques received either unadjuvanted H1 stem v2 or H1 stem v2 adjuvanted with SLA-LSQ containing SLA and QS-21 in a ratio of 5:2 μg, 10:25 μg, 25:25 μg or 50:50 μg, H1 stem v2 adjuvanted with AS01_B_ containing 50:50 μg MPL^®^ and QS-21 or Al(OH)_3_ + NaCl (*N* = 8–9) in a 3-dose immunization regimen on week 0, 8 and 34. CD4 T cell responses were analyzed with ICS at study week 10 (2 weeks post second immunization) and 36 (2 weeks post third immunization). The median frequency of CD4 + CD69+ T cells stimulated with full-length (FL)-HA or HA stem peptide pools expressing IFN-γ, IL-2 or TNF-α are shown after subtraction of the percent positive cells in the DMSO-stimulated (mock control) group. Single positive (SP), double positive (DP), triple positive (TP). Comparisons between the SLA-LSQ- and Al(OH)_3_ + NaCl-vaccines groups with the AS01_B_ group were performed by a Wilcoxon rank sum test. Statistical differences are indicated by asterisks: *P < 0.05, **P < 0.01, ***P < 0.001. Additional statistical comparisons are shown in Supplemental Tables 6–9. Responses in individual animals are shown in Supplemental Figs. 6, 7. Flow cytometry controls are shown in Supplemental Fig. 8.

T cell responses were also evaluated at week 10 (2 weeks post dose 2) and week 36 (2 weeks post dose 3) by intracellular cytokine staining after stimulation of PBMCs with either a full-length (FL)-HA or a HA stem peptide pool. Overall, we found low frequencies of CD4 and CD8 T-cells positive for IFN-γ, IL-2 or TNF- α, with the highest frequencies of cytokine-positive CD4 T-cells detected in the AS01_B_ group both at week 10 and week 36 (Fig. 6, Supplemental Figs. 5–8).

In addition, antigen-specific cytokine secretion was measured at week 36 using a multiplex assay. The standardized group means in cytokine-positive T-cell subsets as measured by ICS and cytokine/chemokine secretion by Luminex after stimulation with full-length HA and HA stem are shown. Overall, the highest fold change in cytokine-positive T-cells and secreted cytokines and chemokines was detected in the H1 stem v2 group adjuvanted with AS01_B_, showing a proinflammatory cytokine profile, similar to what has been observed before with AS01_B_ in blood^39^ (Supplemental Fig. 9).

## Discussion

A broadly protective influenza vaccine is required to improve the protection against seasonal influenza and to provide protection against potentially pandemic influenza strains that may originate from avian and swine influenza viruses^40^. We developed a subunit vaccine consisting of a stabilized trimeric influenza A group 1 HA stem antigen^7–9^, capable of generating broadly neutralizing antibodies. We further improved the original H1 stem vaccine^7^ by incorporating two additional mutations (Y392P/R and E434Q), that increase its expression and trimer stability without affecting antigenicity and protective potency, resulting in H1 stem v2.

We and others have previously shown that robust HA stem-specific antibody responses can be induced in naïve and pre-exposed NHP^7,41–44^, with increased antibody levels in the presence of adjuvants in naïve animals^42,43^. Although NHP lack the IGHV1-69 allele (prevalent in many human group 1 HA stem antibodies) or orthologs hereof, group 1, group 2 and cross-reactive HA stem-specific antibodies were efficiently induced in vaccinated NHPs showing alternative immunoglobulin encoding usages^43,44^, supporting the use of NHP in the evaluation of HA stem vaccines. Here, we evaluated the immunogenicity of H1 stem v2 with the adjuvants Al(OH)_3_ + NaCl, AS01_B_ and SLA-LSQ. H1 stem v2 adjuvanted with AS01_B_ and 50:50 μg SLA-LSQ (which both contain 50 μg QS-21 and 50 μg MPL^®^ or SLA) induced comparable broadly protective HA stem binding and neutralizing antibody titers, which suggests that the bacterium-derived MPL^®^ and the synthetic TLR4 agonist SLA have comparable adjuvanting properties in NHP. We did not directly evaluate the protective capacity of the H1 stem v2-induced antibodies in NHP. However, we have previously shown that H1 stem v1 antibodies induced in NHP can both directly protect against pandemic H1N1 challenge^7^ and indirectly after passive immunization of mice with serum from H1 stem v1-immunized NHP followed by H1N1 and H5N1 challenge^9^. Given that the protective capacity between the H1 stem v1 and the H1 stem v2 is highly similar in mice, in our opinion the protective capacity of the H1 stem v2-induced antibodies in NHP is also identical to H1 stem v1.

Although the AS01_B_-adjuvanted herpes zoster (*Shingrix*) vaccine is highly effective^15,16^, the strong immunostimulatory properties are also linked to increased reactogenicity^39^, such as systemic and injection site reactions. For example, solicited or unsolicited severe grade 3 reactogenicity were reported for 10% of the herpes zoster (*Shingrix*) vaccine recipients^15,16^. Lower reactogenicity has been observed using a Respiratory Syncytial Virus Prefusion F protein adjuvanted with AS01_E_ (25 μg MPL^®^ and 25 μg QS-21), compared with AS01_B_ (50 μg MPL^®^ and 50 μg QS-21)^45^. Reducing the dose of SLA and QS-21 might therefore be an approach to reduce potential adjuvant-driven reactogenicity in humans. However, we did not detect local-injection site reactions using Draize scoring in NHPs with any of the adjuvant formulations, indicating that different readouts or animal models are needed to evaluate the local reactogenicity. It appears that a composite of biomarkers, in particular, IL-6, CRP, and for highly immunogenic products, the IFN-signalling pathway, are linked to systemic reactogenicity^46^. Although we did not measure these cytokines directly in whole blood, antigen-specific restimulation of PBMCs results in lower levels of those cytokines for the SLA-LSQ formulation compared to AS01_B_, suggesting that systemic reactogencity might be lower. A malaria vaccine with as little as 1 μg of the synthetic TLR4 agonist Glucopyranosyl Lipid Adjuvant (GLA) and 0.4 μg QS-21 in a liposomal formulation has been shown to be immunogenic in humans^47^. In addition, clinical trials are currently performed with 5–10 μg GLA and 2–4 μg QS-21 (NCT03589794, NCT02508376, and NCT04177355). However, H1 stem protein formulated with 5:2 μg SLA-LSQ induced significantly lower HA stem binding and neutralizing antibody titers in NHPs, compared with AS01_B_. While the lower adjuvant concentration may contribute to the reduced immunogenicity of this formulation, we hypothesize that the presence of larger fused or aggregated liposomes in this formulation also negatively impacts immunogenicity as fewer particles in the preferred size range are present. In contrast, H1 stem v2 formulated in SLA-LSQ with as low as 10 µg SLA and 25 µg QS-21 induced antibody titers largely comparable with the AS01_B_-adjuvanted group, while these formulations with a lower SLA and QS-21 dose may have the potential to reduce reactogenicity in humans.

Aluminum-based adjuvants also provoke considerably less reactogenicity in humans compared with AS01_B_^12,39^, but this is often accompanied by a reduction in immunogenicity^12,39,48^. However, aluminum hydroxide-based commercial vaccine formulations do in general not combine low amounts of Tris buffer with relatively high amounts of NaCl. We found that two vaccinations with H1 stem protein adjuvanted with an optimized aluminum hydroxide [Al(OH)_3_ + NaCl] formulation induced HA stem-binding and neutralizing antibodies that were comparable with two vaccinations with H1 stem protein adjuvanted with AS01_B_. We demonstrated in mice that formulation with Al(OH)_3_ + NaCl induced stronger immunogenicity and vaccine efficacy, compared with Al(OH)_3_. Although the percentage of bound H1 stem v2 was comparable in the Al(OH)_3_ and Al(OH)_3_ + NaCl formulations, NaCl is thought to improve the immunogenicity by increasing particle aggregation^32^.

We detected the highest cellular responses upon vaccination with H1 stem protein adjuvanted with AS01_B_. SLA-LSQ induced lower cellular responses, even at TLR4 agonist and QS-21 concentrations similar to AS01_B_. This might suggest that a synthetic TLR4 agonist less efficiently activates T-cell responses than bacterium-derived MPL^®^. T-cell responses appeared to be higher upon stimulation with an HA stem peptide pool, compared with a FL-HA peptide pool both at week 10 and week 36. A possible explanation could be the size and complexitiy of the peptide pools (and associated DMSO concentration), which is around twice the size for the FL-HA pool. It has been shown for CD8 T cells that peptide pool size can influence responses in assays^49,50^. Another explanation is that there might be a response to neoepitopes present in HA stem proteins. T cell epitope mapping in NHP would be required to verify this. As T cell responses were low in general, and H1 stem appears to contain a low number of (protective) T cell epitopes^51^, further characterization of potential enhancement of the T cell response by SLA-LSQ with an antigen known to induce robust T cell responses is required.

We observed a slow decline in HA stem-specific antibody levels after the 2nd dose independent of the adjuvant used. Others have reported different observations in NHP, varying from a strong decline to an increase of antibody levels over time^42,43,51–53^, but a direct comparison is difficult, as studies differ in many aspects such as antigen used, infection versus vaccination, use of adjuvants and follow-up time, among others. In addition, we report here that a 3rd vaccination to mimic responses in a pre-exposed population boosted HA stem-specific antibody levels. This was also reported for an adjuvanted comparable HA stem protein immunogen in NHPs^43^. AS01_B_ and SLA-LSQ starting at 10 µg SLA and 25 µg QS-21 boosted antibody titers to a higher level than Al(OH)_3_ + NaCl post dose 3, suggesting that AS01_B_ and SLA-LSQ recall B-cell responses more efficiently. Further characterization of the antigen-specific B and plasma cell populations, antibody repertoire and antibody characteristics would be required to confirm this observation. In humans, AS01_B_ induced high-avidity antibodies at a higher frequency upon antigen recall, suggesting that AS01_B_ contributes to affinity maturation^54^. By contrast, immunization of NHP with a HA stem antigen in a live virus pre-exposure setting did not boost HA stem-specific antibody levels^52^, which could be explained by the fact that no adjuvant was used in this study and was also recently observed in humans, where a second dose of an unaduvanted nanoparticle HA stem antigen dit not further boost responses^55,56^. This emphasizes the benefits of adjuvants for induction or boosting of HA stem-specific immune responses. In our NHP study, levels of HA stem-specific antibodies were higher after the 3rd vaccination when directly compared with stem-HA antibody titers in the adult (pre-exposed) human population after seasonal influenza vaccination using the same ELISA assay to measure samples. This suggests that our H1 stem-based vaccine has the potential to boost HA stem antibody titers in humans.

Overall, we have shown that an H1 stem protein adjuvanted with various adjuvants durably enhanced the HA stem-specific humoral immune response in an animal model relevant to humans. All adjuvant formulations elicited comparable humoral responses in vivo, except for the lowest SLA-LSQ dose, in line with in vitro formulation findings. These data support an ongoing phase 1/2a clinical trial in which the safety, reactogenicity and immunogenicity of H1 stem v2 (INFLUENZA G1 mHA, NCT05901636) is being evaluated in healthy adults.

## Methods

### Human sera

Serum was obtained from a vaccine safety and immunogenicity trial (INF-V-A017, EudraCT #2012-001693-28)^35^, in which subjects received Inflexal V, a trivalent seasonal influenza vaccine of the 2011/2012 composition (H1N1 A/California/07/2009, H3N2 A/Victoria/210/2009 and B/Brisbane/60/2008). Blood was collected on the day of vaccination and four weeks after vaccination. The study was approved by the ‘Universitair Ziekenhuis Antwerpen Comité voor Medische Ethiek’ ethical review board. Written informed consent was obtained from all subjects prior to enrolment.

### Vaccines and adjuvants

DNA encoding tag-free H1 stem (H1 stem v1, design and alignment with full-length HA amino acid sequence as described by Impagliazzo et al.^7^) and H1 stem v2, optimized for expression and manufacturability, were synthesized and cloned into pcDNA3 DNA plasmid with a modified cytomegalovirus promotor (Genscript). All H1 stem proteins were based on the HA sequence from H1N1pdm A/California/07/2009, except for the H1 stem v1 used in the murine challenge experiment which was based on H1N1 A/Brisbane/59/2007 HA sequence and also known as ‘#4900”^7^. Recombinant protein was transiently expressed in ExpiCHO cells.

H1 stem v2 has 31% amino acid identiy similarity with full length H1N1 A/Brisbane/59/2007 HA, while the H1N1 A/Brisbane/59/2007 HA stem domain has 67% amino acid identiy similarity with H1 stem v2. H1 stem v2 has 28% amino acid identiy similarity with full length H5N1 A/Hong Kong/156/1997 HA, while the H5N1 A/Hong Kong/156/1997 HA stem domain has 61% amino acid identiy similarity with H1 stem v2.

### Expression titers and trimer content of recombinant H1 stem protein

Expi293F culture supernatants were harvested at day 3 post transfection, clarified by 0.22 µm filtration. Analytical SEC at day of harvest was performed on an ultra-high-performance liquid chromatography system (Vanquish, Thermo Fisher Scientific) and µDAWN light Scattering detector (Wyatt) coupled to an Optilab µT-rEX Refractive Index Detector (Wyatt). The cleared cell culture supernatants were applied to a Unix-C SEC-300 15 cm column (Sepax) with the corresponding guard column (Sepax) equilibrated in running buffer (150 mM sodium phosphate, 50 mm NaCl, pH 7.0) at 0.3 ml/min. Analytical SEC data was analyzed using the Chromeleon software package (Thermo Fisher Scientific).The trimer content was calculated as the percentage of total protein based on peak heights (in mAU) of the monomeric and trimeric species.

### Purification of recombinant H1 stem protein

H1 stem v1 and v2 were purified from the clarified ExpiCHO culture supernatant, harvested at day 7, by a two-step protocol. First H1 stem protein was captured by affinity chromatography using a resin consisting of an H1 stem protein binding single domain immobilized to POROS beads (Thermo Fisher Scientific). Following elution by application of a step gradient of elution buffer (0.1 M TRIS, 2 M MgCl_2_, 40% propylene glycol, pH 7.4), the antigen containing fractions were pooled and further purified by size exclusion chromatography (Superdex 200 pg 2.6 × 60 cm) using 20 mM TRIS, 150 mM NaCl, pH 7.8 as mobile phase.

### In vitro H1 stem protein characterization

Thermal stability of purified H1 stem protein was assessed by Differential Scanning Fluorimetry (DSF). Melting temperatures were determined by monitoring the fluorescent emission of Sypro Orange Dye (Thermo Fisher Scientific) added to 6 µg of protein in solution. Starting at 25 °C, the temperature was increased at a rate of 54 °C per hour to a final temperature of 95 °C and melting curves were measured using a ViiA7 real-time PCR machine (Applied Biosystems), and Tm_*50*_ values were derived from the negative first derivative. Protein antigenicity was evaluated by enzyme-linked immunosorbent assay (ELISA). H1 stem protein was directly coated in half area 96-well plated (0.25 µg/mL) and incubated with mAb CR9114^28^, CT149^29^ (10 µg/mL starting concentration), and Fc fused SD38^30^ (4 µg/mL starting concentration) in a 3-fold dilution series following incubation with an anti-human Fc horseradish peroxidase secondary antibody (mouse anti-human IgG, Jackson ImmunoResearch, cat 109-035-098, 3750-fold diluted). The luminescent signal of bound antibodies was determined following substrate addition (BM Chemiluminescence ELISA Substrate (POD), Roche) and the EC_50_ values of the S-curves were determined as an average of 3 independent assays.

### Adjuvants

Aluminum phosphate [AlPO_4;_ Adju-Phos™] and aluminum hydroxide [Al(OH)_3_: Alhydrogel™ 2%] adjuvants were manufactured and supplied by Croda Denmark, formerly known as Brenntag Biosector, Federikssund, Denmark. The amount of adsorbed protein was indirectly defined by measuring the residual protein in the supernatant after centrifugation of the protein-adjuvant mixture^57^. The liposomal adjuvants, SLA-LSQ, were manufactured and supplied by Access to Advanced Health Institute (AAHI, Seattle, USA), formerly known as Infectious Diseases Research Institute (IDRI), using QS-21 purified from *Quillaja saponaria* bark extract as previously reported^58^. The AS01_B_ adjuvant (GlaxoSmithKline Biologicals, Rixensart, Belgium) was obtained from the adjuvant vial from the Shingrix vaccine (i.e., without mixing in lyophilized glycoprotein E from the separate antigen vial).

### In vitro characterization of SLA-LSQ

SLA-LSQ stock solutions were mixed 1:1 with the formulation buffer (10 mM Tris, 2.5% sucrose, 150 mM NaCl), either with or without H1 stem protein, and characterized by absorbance (turbidity at 350 nm) and fluorescence emission spectroscopy (excitation 280 nm, emission 340 nm) using a Biotek Neo plate reader. In addition, dynamic light scattering was evaluated using a Wyatt Dynapro II plate reader. After characterization, Nile Red was added and mixed into the formulations and the fluorescence emission at 635 nm was measured after excitation at 550 nm. The SLA-LSQ formulations were also characterized by fluorescence microscopy, using 10 µl in a MultiCount 10™ microscopy slide, according to the staining method described by Demeule et al.^59^. Zeta-potential (mV) was determined using a Zetasizer Nano (Malvern). Nanoparticle tracking analysis (NTA) was measured using a Nanosight NS300 (Malvern) with a flowcell. To visualize the individual particles in NTA, formulations required a dilution of 300-times in 10 mM Tris, 2.5% sucrose and 150 mM NaCl. Video capture was performed during 120 s.

### Mouse studies

Mouse experiments were approved by the Dutch Central Authority for Scientific Procedures on Animals (Centrale Commissie Dierproeven) and conducted in accordance with the European guidelines (EU directive on animal testing 2010/63/EU and ETS 123) and local Dutch legislation on animal experiments. The in vivo phase of mouse studies was performed at Triskelion, Zeist, The Netherlands. Female BALB/cAnNCrl mice aged 6–8 weeks at the start of study were purchased from Charles River Laboratories (Germany). Mice were vaccinated intramuscularly with 100 μl (divided over the two hind legs) H1 stem v2 vaccine adjuvanted with either 50 μg 2% AlPO_4_, Al(OH)_3_, Al(OH)_3_ + PBS or Al(OH)_3_ + NaCl. The characteristics of the aluminum salt-based adjuvant formulations are listed in Table 1. Blood was collected via the tail vein. Immunizations and blood collections were performed on conscious, restrained animals. Mice were intranasally inoculated with 12.5xLD50 of H1N1 A/Brisbane/59/2007 or H5N1 A/Hong Kong/156/1997 in a volume of 50 µl (25 µl per nostril) under general anesthesia with ketamine/xylazine applied intraperitoneally. Health monitoring was performed twice daily during the 21-day follow-up after virus inoculation. Animals were considered moribund and euthanized for ethical reasons if lethargy is noted in four subsequent observations. The surviving animals were sacrificed on day 21 by O_2_/CO_2_ anesthesia followed by CO_*2*_ asphyxiation.

### NHP study

Housing and handling of NHPs was performed in accordance with the standards of the AAALAC International’s reference resource: the 8^th^ edition of the Guide for the Care and Use of Laboratory animals, Animal Welfare Act as amended, and the 2015 reprint of the Public Health Service (PHS) Policy on Human Care and Use of Laboratory Animals. Handling of samples and animals occurred in compliance with the Biosafety in Microbiological and Biomedical Laboratories (BMBL), 5th edition (Centers for Disease Control). This study was performed under IACUC-approved protocol no. 18-19.

Fifty-seven adult (28 male and 29 female) Cynomolgus monkeys (*Macaca fascicularis*) were purchased from Beijing Prima Biotech (China) and the in vivo phase of the study was performed at Alpha Genesis Inc, Yemassee, South Carolina. Prior to study start, all animals were subjected to a health assessment and they were confirmed negative for tuberculosis, simian immunodeficiency virus (SIV), simian retrovirus (SRV), simian T-lymphotropic virus (STLV), Herpes B virus, measles and seronegative for influenza viruses as measured by a haemagglutinin inhibition assay (HI assay) for influenza strains known to have been recently circulating in the human population prior to study start. The animals were assigned to 7 study groups using randomized stratification based on body weight and sex.

Groups of 8–9 influenza-naïve cynomolgus macaques were intramuscularly immunized with 50 µg H1 stem v2 protein. The antigen was either unadjuvanted (Group 1) or mixed with SLA-LSQ adjuvant formulations that differ in the final amount of SLA and QS21: 50 µg SLA/50 µg QS-21 (Group 2), 25 µg SLA/25 µg QS-21 (Group 3), 10 µg SLA/ 25 µg QS-21 (Group 4), 5 µg SLA/ 2 µg QS-21 (Group 5). Two groups were immunized with either H1 stem v2 adjuvanted with 750 µg aluminum hydroxide adjuvant [Al(OH)_3_ + NaCl, Group 6] or human dose of AS01_B_ (50 µg MPL^®^:50 µg QS-21; Group 7). Animals were immunized through the intramuscular route in week 0, week 8 and week 34 in an injection volume of 0.75 mL. Blood for serum was obtained at regular intervals. One animal from group 7 (AS01_B_) was lost to follow-up as it did not recover from anesthesia after biotechnical procedures in week 12. After reviewing of all data, it was decided that the death was deemed unrelated to vaccine or adjuvant. For all procedures, the animals were anesthetized intramuscularly with ketamine hydrochloride (10–20 mg/kg) or tiletaminezolazepam (5–8 mg/kg). The injection site was marked with indelible ink and scored according to the Draize Grading scale for 3 days post immunization, where edema was graded as none (no swelling), minimal (slight swelling), mild (defined swelling—distinct), moderate (defined swelling— raised), severe (pronounced swelling). Erythema was graded as none (normal color), minimal (light pink), mild (bright pink/ pale red), moderate (bright red), severe (dark red).

### Mouse ELISA

HA-specific binding IgG antibody levels were measured by ELISA against a H1N1 A/California/07/2009 full-length HA. Briefly, 96-well ½ area plates were coated for 2 h with 0.5 µg/ml H1 A/California/07/2009 HA protein (Protein Sciences) at 37 °C in a humidified incubator. Plates were washed with PBS with 0.05% Tween (PBS-T) and subsequently blocked with block buffer (PBS + 2% BSA). After incubation for 1 hour, the plates were washed with PBS-T and serum/ control samples were added to the plates in duplicate, serially diluted in block buffer. Following a wash with PBS-T, a 1:20.000 dilution of anti-mouse IgG-HRP (KPL cat 474-1802) was added, incubated for 1 h, washed with PBS-T and Enhanced ChemiLuminescence (ECL, Bio-Rad) detection substrate was added. The luminescence signal was determined after 10 min, expressed as relative luminescence units (RLU). RLU signals were normalized per plate, and the log10 transformed normalized values were fitted using a four-parameter logistic model. Titers were calculated as a dilution ratio between a sample and the reference standard at 50% of the fitted signal (log10 relative titer).

### NHP and human ELISA

HA-specific binding IgG antibody levels were measured by ELISA against C-terminally biotinylated H1N1 A/California/07/2009 full-length HA and a C-terminally biotinylated H1N1 A/California/07/2009 HA stem with different engineered stabilization domains^60^ to exclude irrelevant cross-reactive responses to non-HA derived epitopes. Briefly, 96-well ½-area plates were incubated with 5 μg/mL streptavidin for 2 h at 37 °C in a humidified incubator. Plates were washed with PBS with 0.05% Tween (PBS-T) and subsequently blocked with block buffer (PBS + 1% casein). Plates were incubated with 1 µg/ml C-terminally biotinylated H1N1 A/California/07/2009 full-length HA or HA stem. After incubation for 1 h, serum samples or 3 µg/ml reference standard (mAb CR9114^28^) were added to the plates in duplicate, serially diluted in block buffer and incubated for 1 h. Following a wash with PBS-T, a 1:3750 dilution of anti-human IgG-HRP (Jackson ImmunoResearch, cat 109-035-098) was added, incubated for 1 h, washed with PBS-T and Enhanced ChemiLuminescence (ECL, Bio-Rad) detection substrate added. The luminescence signal was determined after 10 min, expressed as RLU. RLU signals were normalized per plate, and log10 transformed normalized values fitted using a four-parameter logistic model. Titers were calculated as a dilution ratio between a sample and the reference standard at 10% of the fitted signal (log10 relative titer).

### ADCC hFcγRIIIa reporter assay

To measure HA-specific antibody-dependent cellular cytotoxicity (ADCC) Fc effector functions elicited by H1 stem vaccination, serum samples were analyzed using an ADCC hFcγRIIIa reporter assay (Promega). Briefly, human lung carcinoma-derived A549 epithelial cells were maintained in Dulbecco’s Modified Eagle Medium supplemented with fetal calf serum. Two days before the experiment, cells were transfected with plasmid DNA encoding H1N1 A/California/07/2009 full-length HA. One day before the assay, transfected cells were harvested and seeded in 96-well plates. After 24 h, serum samples were diluted in assay buffer and heat-inactivated, followed by serial dilution in assay buffer. The cells were replenished with fresh assay buffer and ADCC Bioassay effector cells (a stable Jurkat cell line expressing human hFcγRIIIa [V158 variant], human CD3γ, and an NFAT-response element driving expression of a luciferase reporter gene) were added and incubated. Bio-Glo Luciferase Assay System substrate was added, and the luminescence signal was read. Data are expressed as endpoint titers, which are defined as the reciprocal dilution where a pre-defined, arbitrarily threshold (5 × 10^4^ RLU) is reached.

### Pseudovirus entry inhibition assay

Neutralizing antibody titers were measured with a pseudo virus particle entry inhibition assay (ppVNA), using a panel of strains expressing influenza A group 1 HA and neuraminidase (NA) at Monogram (San Francisco, USA): H1N1 A/Fort Monmouth/1/1947, H1N1 A/South Carolina/1/1918, H1N1 A/USSR/90/1977, H1N1 A/New Caledonia/20/1999, H1N1 A/Brisbane/59/2007, H1N1 A/California/07/2009, H5N1 A/Hong Kong/156/1997, H5N1 A/Indonesia/05/2005, H6N2 A/Turkey/Massachusetts/3740/1965, H8N4 A/duck/Yangzhou/02/2005, H9N2 A/Hong Kong/1073/1999.

Influenza virus neutralization was measured using a recombinant pseudo-influenza virus assay that involves a single round of infection. A replication-defective retroviral vector (RTV1.F-lucP.CNDO delta U3) containing a firefly luciferase gene was co-transfected into human embryonic kidney (HEK) 293 cell cultures along with HA and NA expression vectors. A fourth expression vector containing a human airway serine protease used in processing the HA protein, TMPRSS2, was also transfected. The resultant pseudoviruses were harvested from culture supernatants, filtered, titrated and stored at −80 °C. Murine leukemia virus envelope (aMLV) pseudotyped virus was used as a control. The pseudovirus stocks, at a concentration giving approximately 30,000-300,000 RLU per well, were incubated at 37 °C for one hour with 3- or 4-fold serial diluted serum samples in a 96-well plate starting at a 1:10 dilution. HEK293 cells were then added to each well and incubated at 37 °C in 5% CO_2_. After 3 days, luciferase substrate and cell lysing reagents were added to the plates which were read on a luminometer. Inhibition curves were fitted by a four-parameter sigmoidal function using nonlinear least-squares and bootstrapping. Resulting curves were used to calculate the serum dilution/antibody concentration required to inhibit virus infection by 50% (IC_50_). Neutralization titers were expressed as the reciprocal of the IC_50_ serum dilution (ID50).

Neutralizing-breadth potency plots were generated according to Joyce et al.^61^. Briefly, the percentage of human-infecting influenza HA subtypes neutralized by serum (defined as percentage coverage) was calculated per animal as the minimum branch length connecting all neutralized viruses (at the specified log10 reciprocal ID50) divided by total branch length of the phylogenetic tree generated from all tested viral strains. The sequence alignment and the phylogenetic tree were generated using the Neighbor Joining method and Jukes-Cantor distance measure using CLC Main workbench 8.1 (Qiagen Aarhus, Denmark). The area under the curve (AUC) of breadth potency plots was calculated using the trapezoidal rule to quantify the breadth of neutralization. Breadth-potency plots were generated and AUC calculations performed using R.4.1.1 and libraries ape 5.6.2^62^ and tidyverse 1.3.1^63^.

### Intracellular cytokine staining (ICS)

Antigen-specific cytokine secretion was measured by ICS at Texas Biomedical Research Institute (San Antonio, Texas). 1–2 × 10^6^ PBMC were stimulated overnight with peptide pools of 15-mer peptides with 11-amino acid overlap (covering the entire length of H1N1 A/California/07/2009 HA or H1 stem), negative control (medium) or positive control (SEB), in the presence of Brefeldin A at 37 °C (5% CO_2_ and 95% humidity). Samples were stained by Live/Dead Aqua dye (Invitrogen) for dead cell discrimination. Cells were surface stained with anti-CD3-PerCP-Cy5.5 (cloneSP34.2, BD cat. 552852, 1:3400 dilution), anti-CD4-APC-H7 (clone L200, BD cat. 560837 1:3400 dilution), and anti-CD8-PE-Cy7 (clone SK1, Biolegend cat. 344712, 1:5667 dilution) for 30 minutes at 4 °C. The cells were then permeabilized with Cytofix/Cytoperm and subsequently stained intracellularly using anti-IFN-γ-APC (clone B27, Biolegend cat. 506510, 1:6000 dilution), anti-TNF-α-FITC (Mab11, BD cat. 552889, 1:2000 dilution), anti-IL-2-BV421 (MQ1-17H12, BD cat. 564164, 1:6000 dilution), anti-CD69-PE (clone FN50, Biolegend cat. 310906, 1:6000 dilution) antibodies for 30 min at room temperature. T cells were identified by consecutive gating on size (lymphocytes; FSC-A versus side scatter-A), single cells (forward scatter [FSC]-H versus FSC-A), live cells, CD3+, CD4+, or CD8+ cells, and CD69+ plus cytokine-positive (Supplemental Fig. 10). Boolean gating was used to measure single, double or triple IFN-γ, TNF-α or IL-2 positive cells. The frequency of cytokine-positive cells is presented after subtraction of the background response detected in the corresponding medium-stimulated sample of each individual animal.

### Luminex

Antigen-specific cytokine secretions were measured with a 14-plex cytokine secretion assay at the Texas Biomedical Research Institute. Briefly, 4 × 10^5^ PBMC were stimulated overnight in the presence of peptide pools of 15-mer peptides with 11-amino acid overlap, covering the entire length of H1N1 A/California/07/2009 HA or H1 stem, negative control (medium) or positive control (SEB). 24 h after stimulation an aliquot of the supernatant was removed to measure concentrations of: IL-22, IP-10, IL-4, Perforin, IFN-y, IL-17, I-TAC, TNF-a, sCD40L, MIG, IL-9, IL-6, MCP-1 and IL-2 by Luminex, according to manufacturer’s instructions. The full-length H1 HA and H1 stem group means have been centered and scaled with the root-mean-squared-error from an analysis-of-variance, with group as factor. Hierarchical clustering has been applied in the resulting heatmap to both the rows and columns. The rows have been sorted within the trees by the mean absolute deviation of the standardized group means, and the columns have been sorted by the mean of the standardized group means. In this way, the more statistically significant assays are at the top and the groups with higher responses are on the right.

### Statistical analysis

#### Mouse study comparing HA stem v1 vs v2

Comparisons of the survival proportion were performed using a two-sided Fisher’s exact test.

#### Mouse study comparing aluminum-based adjuvants

Comparisons of the survival proportion with the mock-group were performed using a two-sided Fisher’s exact test with a 4-fold Bonferroni-correction and a step-wise approach starting with the highest dose of H1 stem per alum salt formulation. Across dose comparisons of the survival proportion were performed using a two-sided Fisher’s exact test with a 4-fold Bonferroni-correction.

Comparisons of the ELISA titers with the mock-group were performed using an ANOVA with treatment as a group factor with a 4-fold Bonferroni-correction and a step-wise approach starting with the highest dose of H1 stem per alum salt formulation. Across dose comparisons of the ELISA titers were performed using an ANOVA with treatment as a group factor with a 4-fold Bonferroni-correction.

#### NHP study

Comparisons between the SLA-LSQ groups and the AS01_B_ group were performed using the Wilcoxon rank sum test for all assays. No multiple comparison adjustment was applied.

#### Human sera

Comparisons were performed using a paired nonparametric Wilcoxon matched-pairs signed rank test.

For all statistical tests, the significance level was 5%. All statistical calculations were done in SAS 9.4 (SAS Institute Inc).

### Supplementary information

Supplemental material

## Data Availability

All data that support the findings of this study are available from the corresponding author upon request.
