# Supplementary material for: Enhancing breadth and durability of humoral immune responses in non-human primates with an adjuvanted group 1 influenza hemagglutinin stem antigen
Source: NPJ Vaccines. 2023 Nov 11;8:176. doi: 10.1038/s41541-023-00772-1 (PMC10640631; doi:10.1038/s41541-023-00772-1)
Supplement: Supplementary file 1 — Supplemental material [file 41541_2023_772_MOESM1_ESM.pdf]

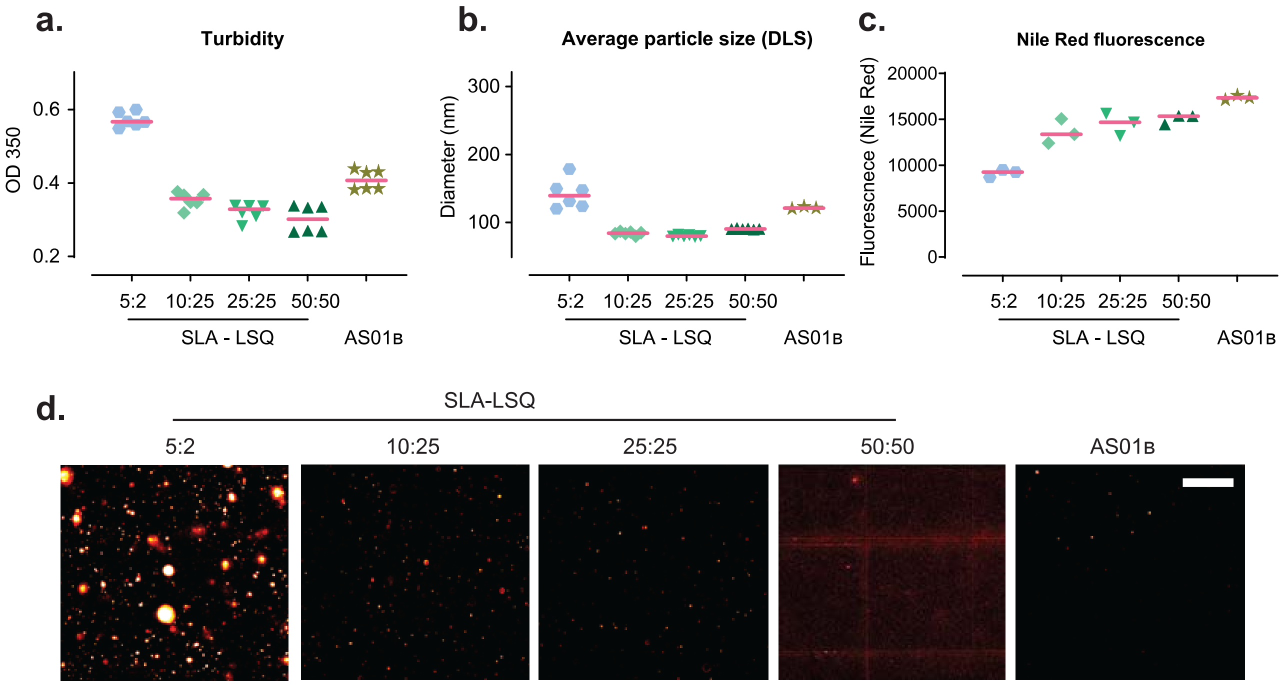

Supplemental figure 1. *In vitro* characterization of SLA-LSQ and AS01$_B$. SLA-LSQ containing SLA and QS-21 in a ratio of 5:2 µg, 10:25 µg, 25:25 µg or 50:50 µg or AS01$_B$ containing 50:50 µg MPL® and QS-21 were characterized without H1 stem v2 protein. **a** Absorption at 350 nm indicates the turbidity. **b** Average particle size was evaluated using dynamic light scattering (DLS). Nile Red fluorescence emission (**c**) provides and indication on the level of interaction of the dye with all hydrophobic particles in solution. Nile Red fluorescence microscopy (**d**) visualizing the larger micrometer-size globular and hydrophobic structures. Scale bar indicates 100 µm.

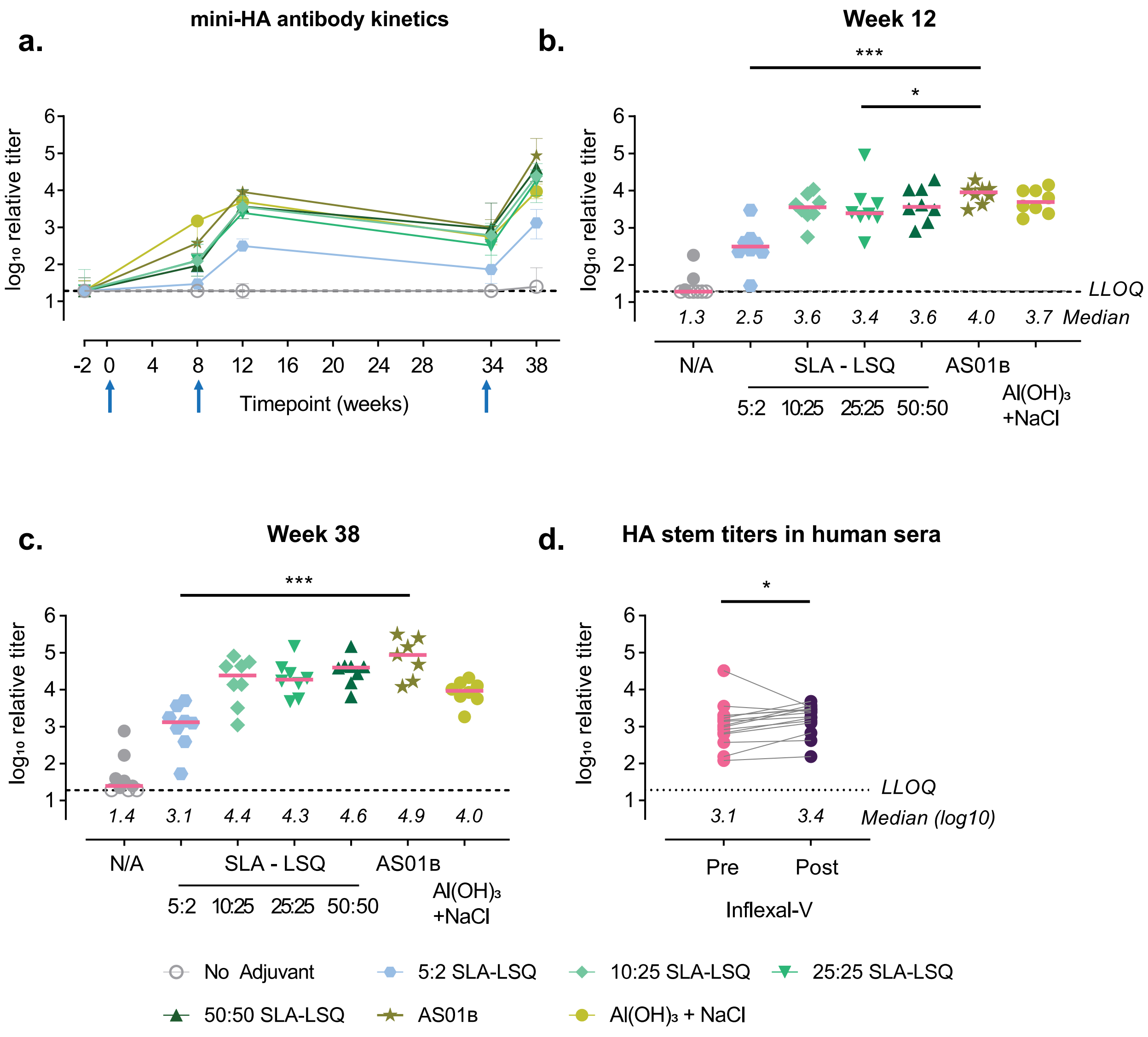

Supplemental figure 2. Influenza HA stem-specific binding antibodies as measured by a HA stem ELISA. a Influenza-seronegative cynomolgus macaques received either unadjuvanted H1 stem v2 or H1 stem v2 adjuvanted with SLA-LSQ containing SLA and QS-21 in a ratio of 5:2 µg, 10:25 µg, 25:25 µg or 50:50 µg, H1 stem v2 adjuvanted with AS01B containing 50:50 µg MPL® and QS-21 or Al(OH)3 + NaCl (N=8-9) in a 3-dose immunization regimen on week 0, 8 and 34. HA stem-specific binding antibody levels were measured at week -2, 8, 12, 34 and 38 using a H1N1 A/California/07/09 HA stem IgG-binding ELISA. Median binding titers per group are shown and error bars denote the interquartile range (IQR). Individual binding titers per animal are shown for week 12 (**b**) and 38 (**c**). Red horizontal bars indicate the group medians and the dotted line indicates the lower limit of quantification (LLOQ). Open symbols indicate the response is at or below the LLOQ. Comparisons between the SLA-LSQ- and Al(OH)3 + NaCl-vaccines groups with the AS01B group were performed by a Wilcoxon rank sum test. Statistical differences are indicated by asterisks: * P<0.05, *** P<0.001. **d** Healthy human volunteers received the seasonal trivalent influenza vaccine Inflexal V that included the influenza strains H1N1 A/California/07/2009, H3N2 A/Victoria/210/2009 and B/Brisbane/60/2008. Blood was collected before vaccination (Pre) and 4 weeks after vaccination (Post). HA stem specific binding antibody levels were measured using a H1N1 A/California/07/09 HA stem IgG binding ELISA. Comparisons were performed using a paired nonparametric Wilcoxon matched-pairs signed rank test.

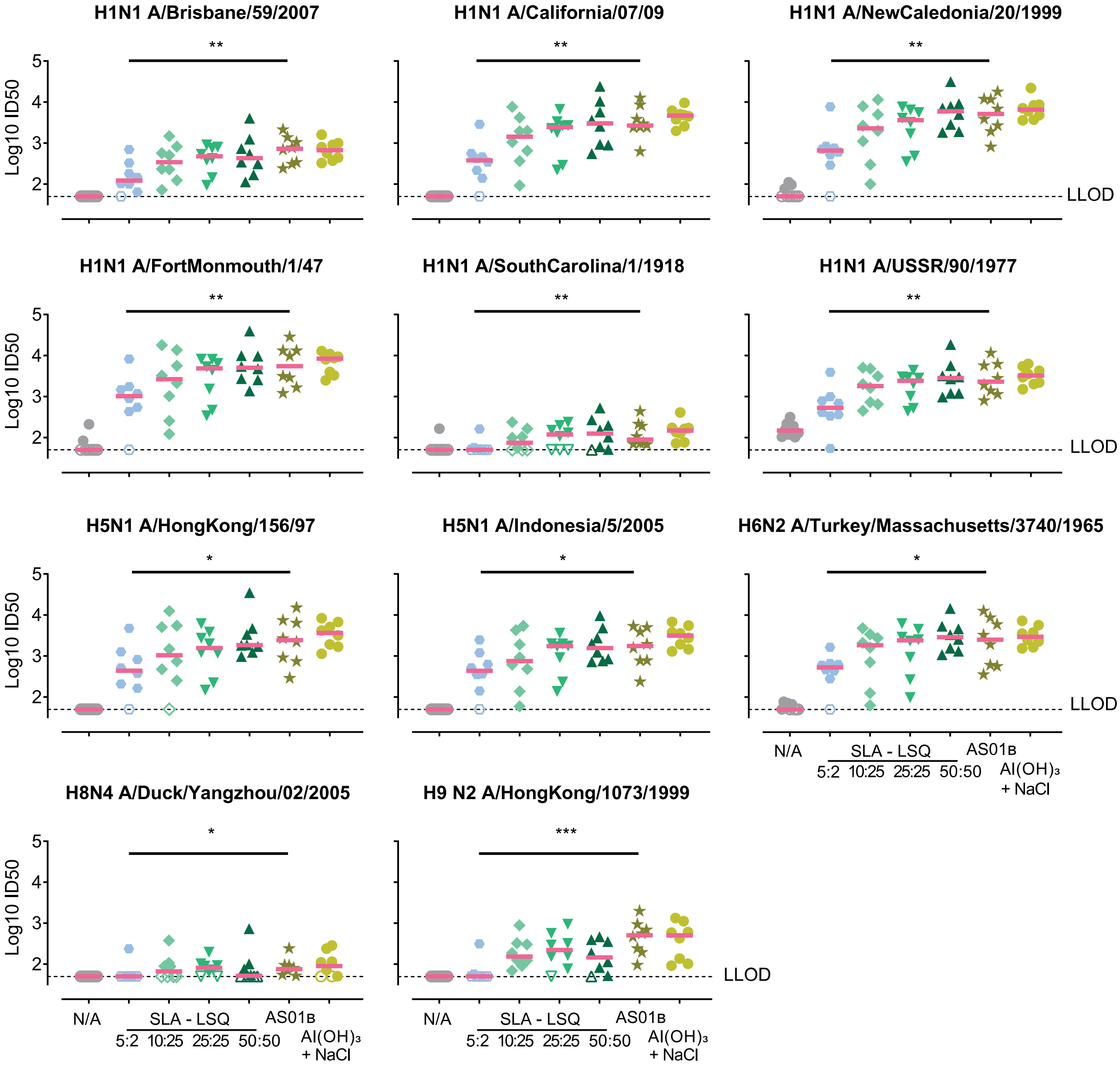

Supplemental figure 3. Neutralizing antibodies induced by vaccination of NHPs with adjuvanted H1 stem protein. Influenza-seronegative cynomolgus macaques received either unadjuvanted H1 stem v2 or H1 stem v2 adjuvanted with SLA-LSQ containing SLA and QS-21 in a ratio of 5:2 µg, 10:25 µg, 25:25 µg or 50:50 µg, H1 stem v2 adjuvanted with AS01B containing 50:50 µg MPL® and QS-21 or $Al(OH)_3$ + NaCl (N=8-9) in a 3-dose immunization regimen on week 0, 8 and 34. Neutralizing antibody titers were measured using a ppVNA against a panel of influenza A group 1 virus strains at week 12 (4 weeks post dose 2). Red horizontal bars indicate the group medians and the dotted line indicates the lower limit of detection (LLOD). Open symbols indicate the response is at or below the LLOD. Comparisons between the SLA-LSQ- and $Al(OH)_3$ + NaCl-vaccines groups with the AS01B group were performed by a Wilcoxon rank sum test. Statistical differences are indicated by asterisks: * $P<0.05$, ** $P<0.01$, *** $P<0.001$.

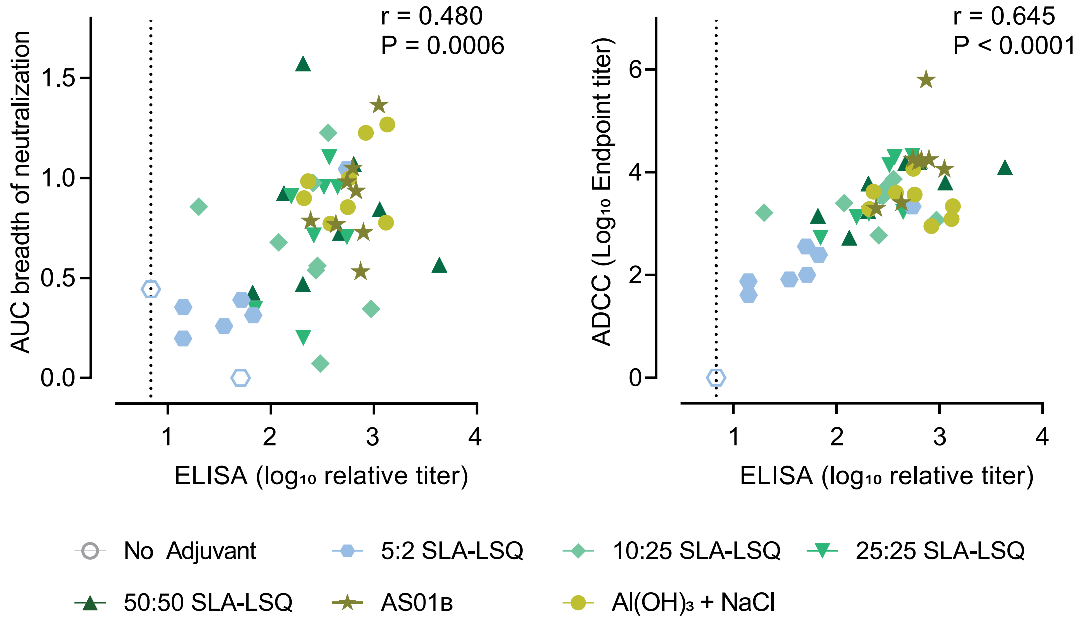

Supplemental figure 4. Antibody titers partially correlate with the breadth of neutralization and ADCC titers. Influenza-seronegative cynomolgus macaques received either unadjuvanted H1 stem v2 or H1 stem v2 adjuvanted with SLA-LSQ containing SLA and QS-21 in a ratio of 5:2 µg, 10:25 µg, 25:25 µg or 50:50 µg, H1 stem v2 adjuvanted with $AS01_B$ containing 50:50 µg MPL® and QS-21 or $Al(OH)_3$ + NaCl (N=8-9) on week 0 and 8. The correlation between ELISA titers with the area under the curve (AUC) for neutralization of breadth-potency curve and ADCC titers at week 12 is shown. Correlation coefficients were calculated using two-sided Spearman rank correlation. The dotted line indicates the lower limit of quantification (LLOQ). Open symbols indicate the response is at or below the LLOQ or an AUC of 0.

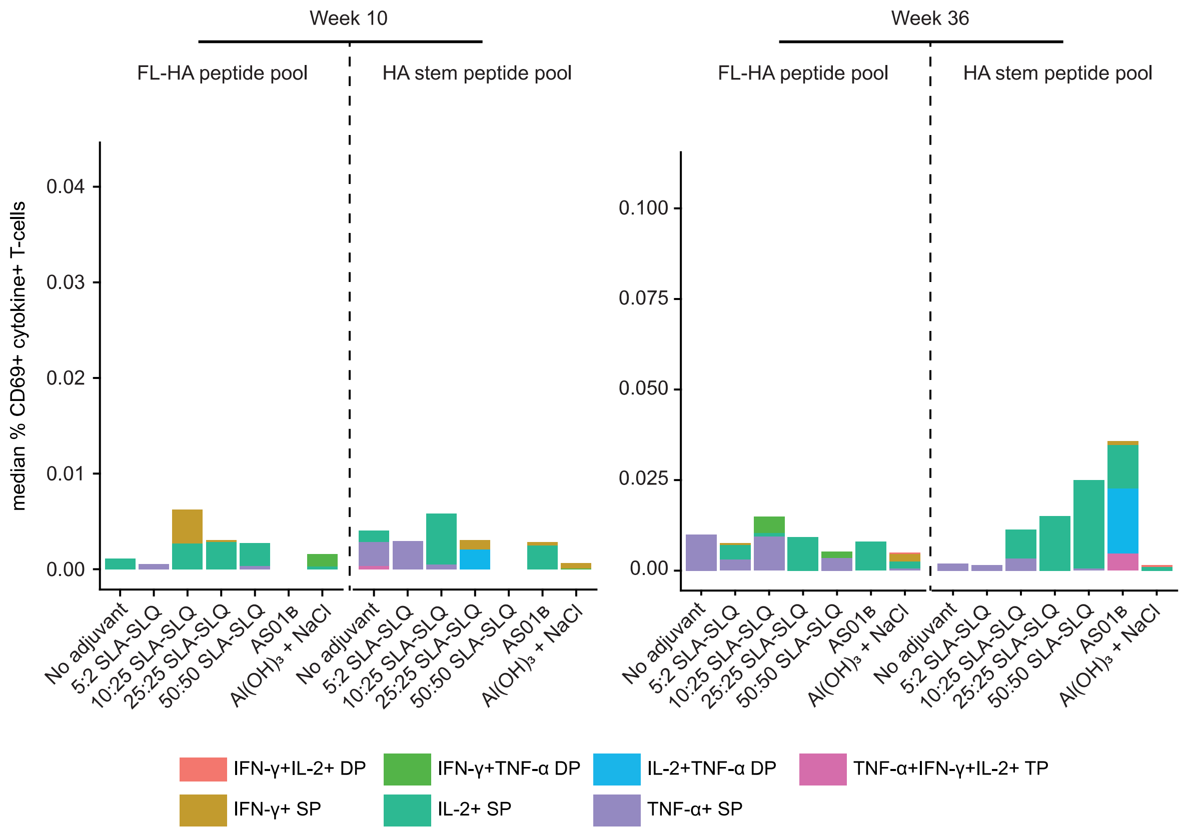

Supplemental figure 5. CD8 responses induced by vaccination of NHPs with adjuvanted H1 stem protein. Influenza-seronegative cynomolgus macaques received either unadjuvanted H1 stem v2 or H1 stem v2 adjuvanted with SLA-LSQ containing SLA and QS-21 in a ratio of 5:2 µg, 10:25 µg, 25:25 µg or 50:50 µg, H1 stem v2 adjuvanted with $AS01_B$ containing 50:50 µg MPL® and QS-21 or $Al(OH)_3$ + NaCl (N=8-9) in a 3-dose immunization regimen on week 0, 8 and 34. CD8 T cell responses were analyzed with ICS at study week 10 (2 weeks post second immunization) and 36 (2 weeks post third immunization). The median frequency of CD8+ CD69+ T cells stimulated with full-length (FL)-HA or HA stem peptide pools expressing IFN-γ, IL-2 or TNF-α are shown after subtraction of the percent positive cells in the DMSO-stimulated (mock control) group. Single positive (SP), double positive (DP), triple positive (TP). Comparisons between the SLA-LSQ- and $Al(OH)_3$ + NaCl-vaccines groups with the $AS01_B$ group were performed by a Wilcoxon rank sum test. Statistical differences are indicated by asterisks: * P<0.05, ** P<0.01, *** P<0.001. Responses in individual animals are shown in **Supplemental figure 6-7**. Flow cytometry controls are shown in **Supplemental figure 8**.

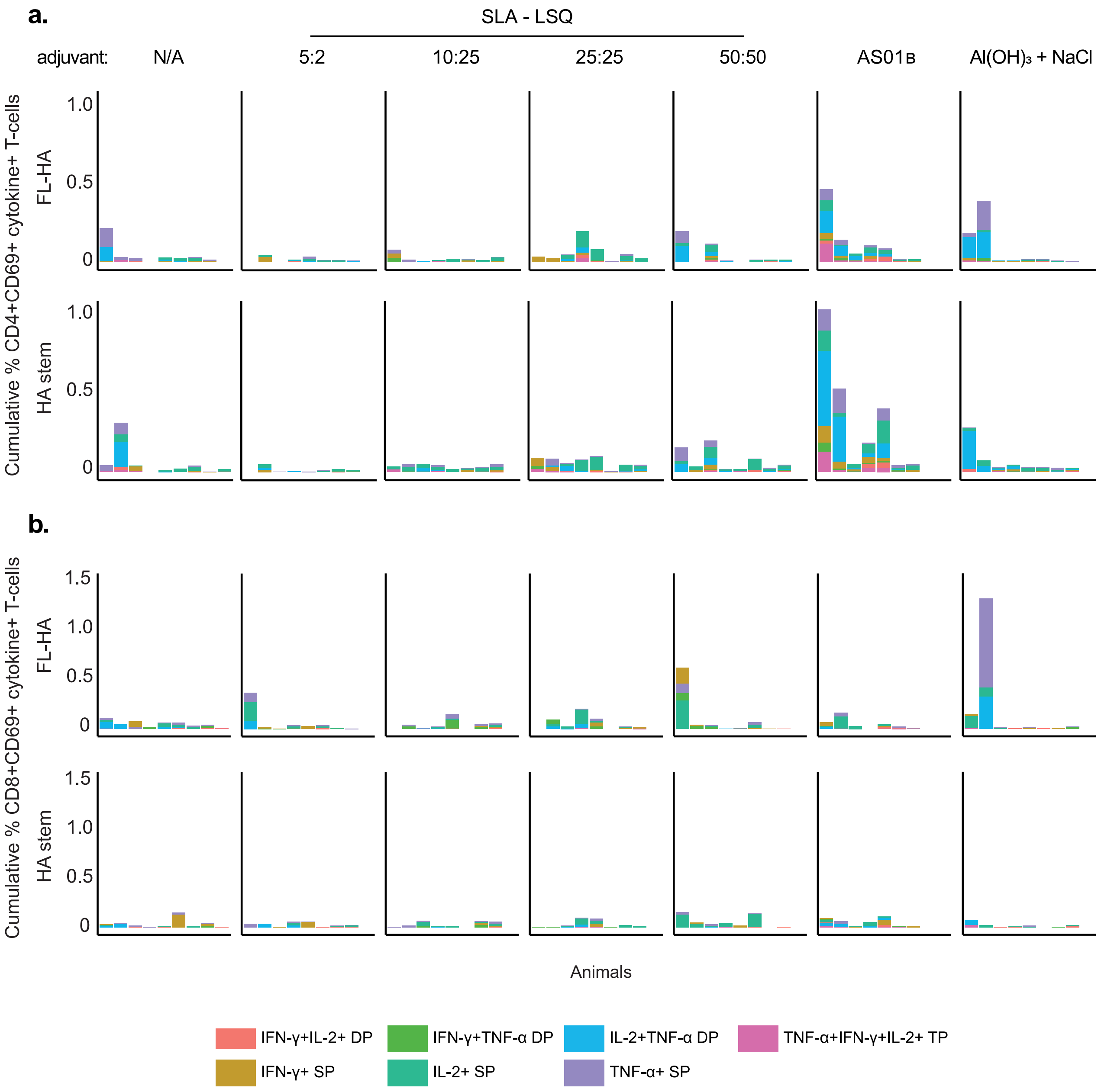

Supplemental figure 6. Cellular responses induced by vaccination of NHPs with adjuvanted H1 stem protein in individual animals week 36. Influenza-seronegative cynomolgus macaques received either unadjuvanted H1 stem v2 or H1 stem v2 adjuvanted with SLA-LSQ containing SLA and QS-21 in a ratio of 5:2 µg, 10:25 µg, 25:25 µg or 50:50 µg, H1 stem v2 adjuvanted with AS01B containing 50:50 µg MPL® and QS-21 or $Al(OH)_3$ + NaCl (N=8-9) in a 3-dose immunization regimen on week 0, 8 and 34. T cell responses were analyzed with ICS at study week 36 (2 weeks post third immunization). The cumulative frequency of CD4+ (**a**) or CD8+ (**b**) CD69+ T cells stimulated with full-length (FL)-HA or HA stem peptide pools expressing IFN-γ, IL-2 or TNF-α are shown after subtraction of the percent positive cells in the DMSO-stimulated (mock control) group. Single positive (SP), double positive (DP), triple positive (TP).

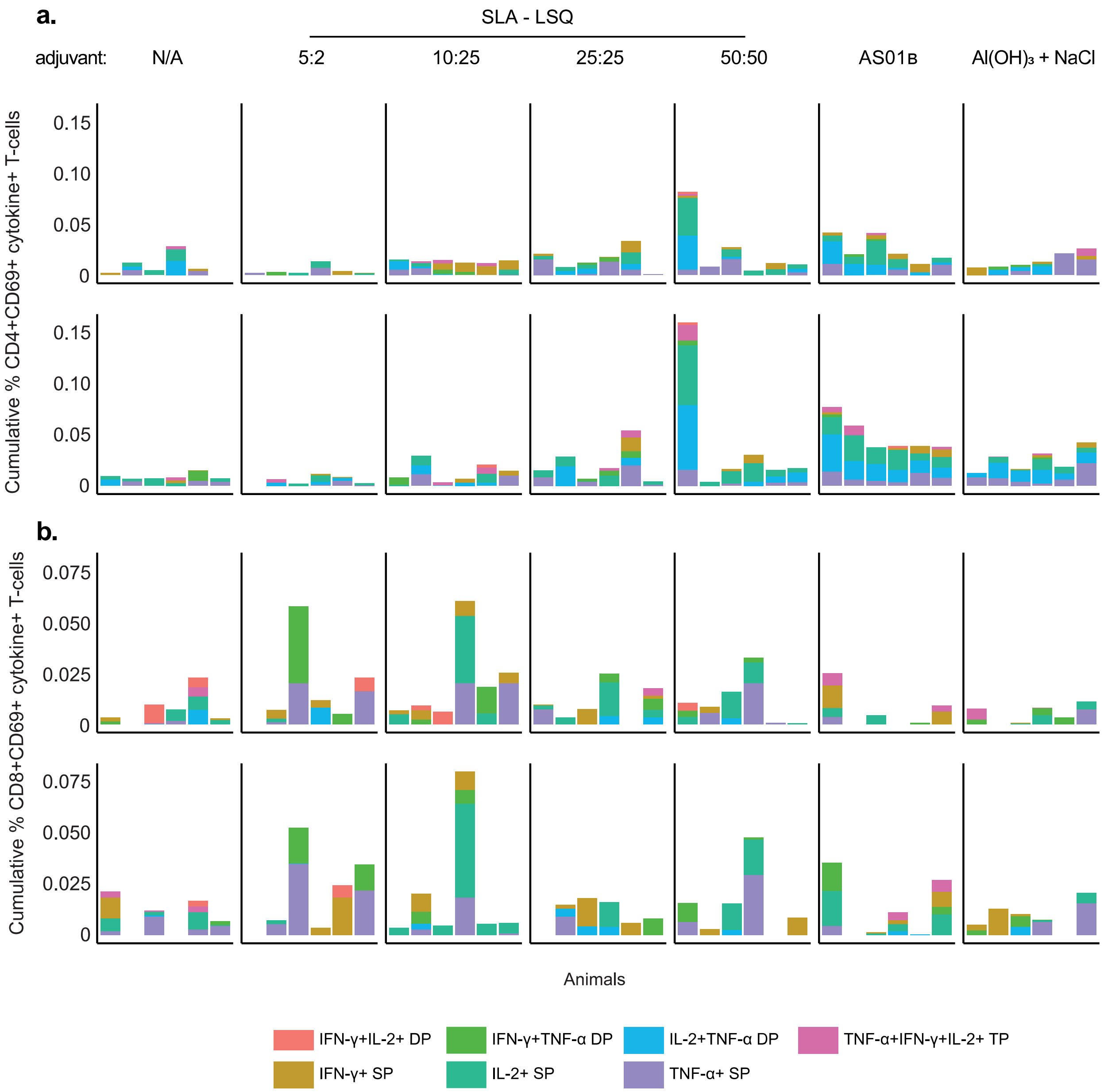

Supplemental figure 7. Cellular responses induced by vaccination of NHPs with adjuvanted H1 stem protein in individual animals week 10. Influenza-seronegative cynomolgus macaques received either unadjuvanted H1 stem v2 or H1 stem v2 adjuvanted with SLA-LSQ containing SLA and QS-21 in a ratio of 5:2 µg, 10:25 µg, 25:25 µg or 50:50 µg, H1 stem v2 adjuvanted with AS01B containing 50:50 µg MPL® and QS-21 or $Al(OH)_3$ + NaCl (N=8-9) in a 3-dose immunization regimen on week 0, 8 and 34. T cell responses were analyzed with ICS at study week 10 (2 weeks post second immunization). The cumulative frequency of CD4+ (**a**) or CD8+ (**b**) CD69+ T cells stimulated with full-length (FL)-HA or HA stem peptide pools expressing IFN-γ, IL-2 or TNF-α are shown after subtraction of the percent positive cells in the DMSO-stimulated (mock control) group. Single positive (SP), double positive (DP), triple positive (TP).

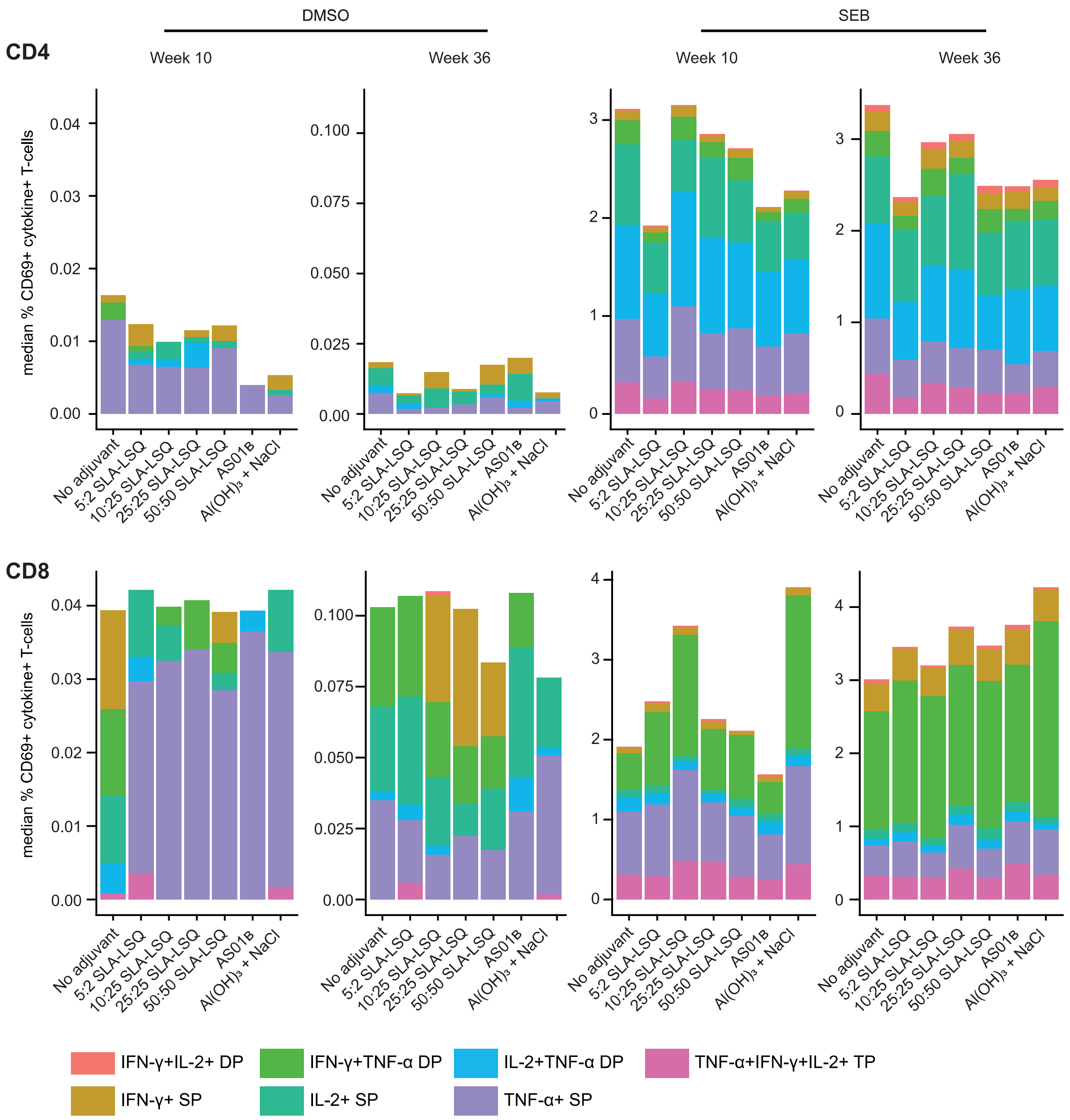

Supplemental figure 8. Flow cytometry controls. Influenza-seronegative cynomolgus macaques received either unadjuvanted H1 stem v2 or H1 stem v2 adjuvanted with SLA-LSQ containing SLA and QS-21 in a ratio of 5:2 µg, 10:25 µg, 25:25 µg or 50:50 µg, H1 stem v2 adjuvanted with $AS01_B$ containing 50:50 µg MPL® and QS-21 or $Al(OH)_3$ + NaCl (N=8-9) in a 3-dose immunization regimen on week 0, 8 and 34. T cell responses were analyzed with ICS at study week 10 (2 weeks post second immunization) and 36 (2 weeks post third immunization). The median frequency of CD4+ or CD8+ CD69+ T cells stimulated with DMSO or SEB expressing IFN-γ, IL-2 or TNF-α are shown after subtraction of the percent positive cells in the DMSO-stimulated (mock control) group. Single positive (SP), double positive (DP), triple positive (TP).

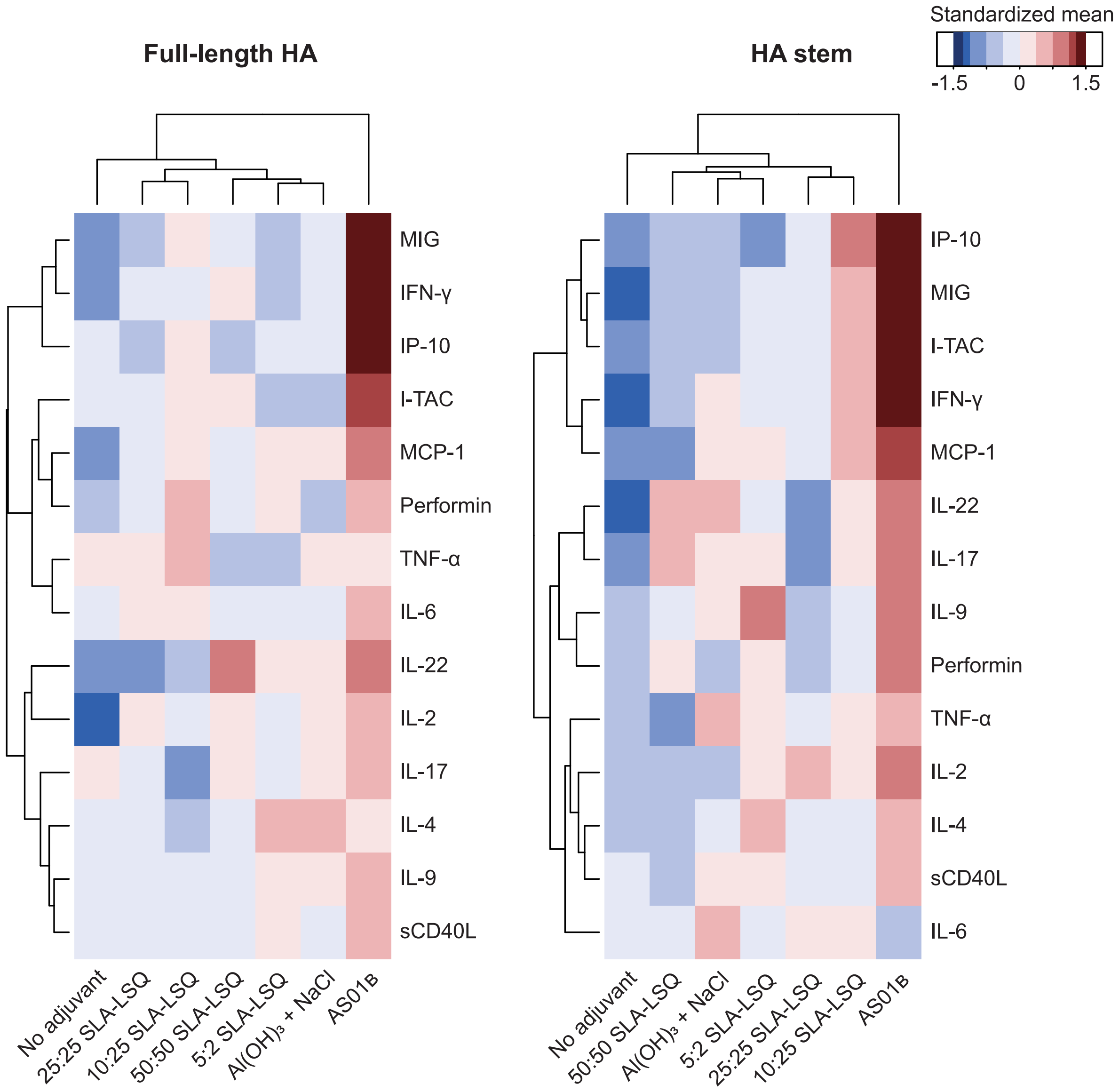

Supplemental figure 9. Cytokine secretion by PBMCs from NHPs vaccinated with adjuvanted H1 stem protein. Influenza-seronegative cynomolgus macaques received either unadjuvanted H1 stem v2 or H1 stem v2 adjuvanted with SLA-LSQ containing SLA and QS-21 in a ratio of 5:2 µg, 10:25 µg, 25:25 µg or 50:50 µg, H1 stem v2 adjuvanted with $AS01_B$ containing 50:50 µg MPL® and QS-21 or $Al(OH)_3$ + NaCl (N=8-9) in a 3-dose immunization regimen on week 0, 8 and 34. PBMCs were collected at week 36 and stimulated with full-length HA or H1 stem protein. Supernatants were analyzed for cytokine secretion after 24 h with a 14-plex cytokine Luminex assay. The centered group means for log Luminex results relative to their ANOVA residual standard deviation are shown in a heatmap with both hierarchical clustering of readout assays and treatment groups.

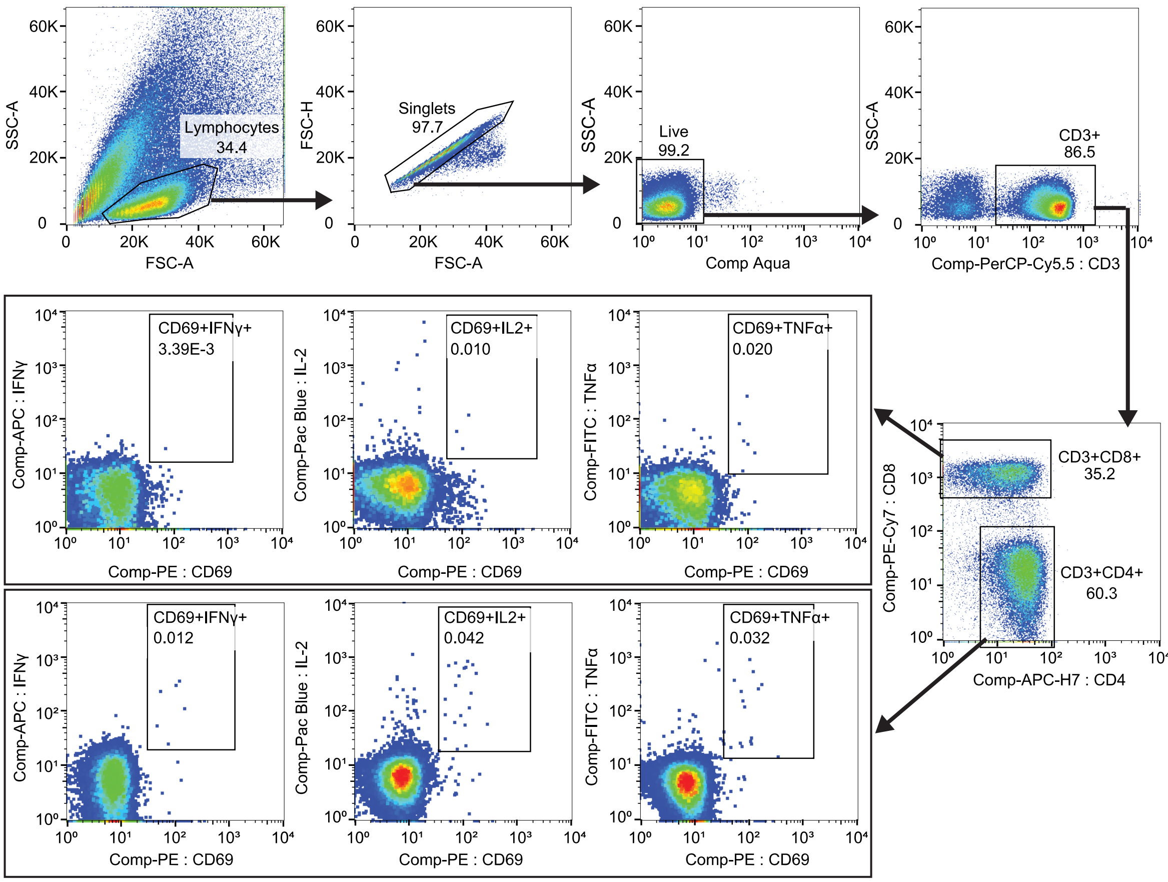

Supplemental figure 10. Gating strategy. T cells were identified by consecutive gating on size (lymphocytes; FSC-A versus side scatter-A), single cells (forward scatter [FSC]-H versus FSC-A), live cells, CD3+, CD4+, or CD8+ cells, and CD69+ plus cytokine-positive.

Supplemental table 1. P-values statistical comparisons antibody titers in a full-length H1N1 A/California/07/09 IgG ELISA week 4. Comparisons between NHP group 1 and group 2-7 were performed by a Wilcoxon rank sum test with 6-fold Bonferroni correction. Comparisons between group 2-7 were performed by a Wilcoxon rank sum test. The direction of the effect is indicated with +/- signs, where +/- p<0.01, ++/-- p<0.01, +++/--- p<0.001.

		group 2	group 3	group 4	group 5	group 6	group 7
Group	Description	SLA-LSQ 50:50	SLA-LSQ 25:25	SLA-LSQ 10:25	SLA-LSQ 5:2	$Al(OH)_3$ +NaCl	$AS01_B$
1	no adjuvant	0.494	1.000	1.000	1.000	<0.001 +++	0.054
2	SLA-LSQ 50:50		0.426	0.713	0.569	0.009 ++	0.267
3	SLA-LSQ 25:25			0.856	0.856	<0.001 +++	0.067
4	SLA-LSQ 10:25				0.856	0.008 ++	0.236
5	SLA-LSQ 5:2					0.005 ++	0.158
6	$Al(OH)_3$+NaCl						0.232

Supplemental table 2. P-values statistical comparisons antibody titers in a full-length H1N1 A/California/07/09 IgG ELISA week 12. Comparisons between NHP group 1 and group 2-7 were performed by a Wilcoxon rank sum test with 6-fold Bonferroni correction. Comparisons between group 2-8 were performed by a Wilcoxon rank sum test. The direction of the effect is indicated with +/- signs, where +/- * p<0.01, ++/-- p<0.01, +++/--- p<0.001.

Group	Description	group 2 SLA-LSQ 50:50	group 3 SLA-LSQ 25:25	group 4 SLA-LSQ 10:25	group 5 SLA-LSQ 5:2	group 6 $Al(OH)_3$ +NaCl	group 7 $AS01_B$
1	no adjuvant	<0.001 +++	<0.001 +++	<0.001 +++	0.002 ++	<0.001 +++	<0.001 +++
2	SLA-LSQ 50:50		0.721	0.645	0.005 --	0.328	0.328
3	SLA-LSQ 25:25			0.798	0.007 --	0.038 +	0.007 ++
4	SLA-LSQ 10:25				0.028 -	0.105	0.038 +
5	SLA-LSQ 5:2					0.001 ++	<0.001 +++
6	$Al(OH)_3$+NaCl						0.878

Supplemental table 3. P-values statistical comparisons antibody titers in a full-length H1N1 A/California/07/09 IgG ELISA week 38. Comparisons between group 1 and group 2-7 were performed by a Wilcoxon rank sum test with 6-fold Bonferroni correction. Comparisons between group 2-8 were performed by a Wilcoxon rank sum test. The direction of the effect is indicated with +/- signs, where +/- * p<0.01, ++/-- p<0.01, +++/--- p<0.001.

Group	Description	group 2<br>SLA-LSQ 50:50	group 3<br>SLA-LSQ 25:25	group 4<br>SLA-LSQ 10:25	group 5<br>SLA-LSQ 5:2	group 6<br>$Al(OH)_3$+NaCl	group 7<br>$AS01_B$
1	no adjuvant	<0.001 +++	<0.001 +++	<0.001 +++	0.002 ++	<0.001 +++	<0.001 +++
2	SLA-LSQ 50:50		0.161	0.645	<0.001 ---	0.007 --	0.694
3	SLA-LSQ 25:25			0.328	<0.001 ---	0.204	0.016 +
4	SLA-LSQ 10:25				0.007 --	0.065	0.613
5	SLA-LSQ 5:2					0.049 +	<0.001 +++
6	$Al(OH)_3$+NaCl						<0.001 +++

Supplemental table 4. P-values statistical comparisons breadth of neutralization. Comparisons between group 1 and group 2-7 were performed by a Wilcoxon rank sum test with 6-fold Bonferroni correction. Comparisons between group 2-8 were performed by a Wilcoxon rank sum test. The direction of the effect is indicated with +/- signs, where +/- * p<0.01, ++/-- p<0.01, +++/--- p<0.001.

Group	Description	group 2<br>SLA-LSQ 50:50	group 3<br>SLA-LSQ 25:25	group 4<br>SLA-LSQ 10:25	group 5<br>SLA-LSQ 5:2	group 6<br>$Al(OH)_3$ +NaCl	group 7<br>$AS01_B$
1	no adjuvant	<0.001 +++	<0.001 +++	<0.001 +++	0.023 +	<0.001 +++	<0.001 +++
2	SLA-LSQ 50:50		0.878	0.505	0.007 --	0.234	0.505
3	SLA-LSQ 25:25			0.645	0.083	0.161	0.382
4	SLA-LSQ 10:25				0.105	0.065	0.195
5	SLA-LSQ 5:2					0.005 ++	0.005 ++
6	$Al(OH)_3$+NaCl						0.442

Supplemental table 5. P-values statistical ADCC reporter assay. Comparisons between group 1 and group 2-7 were performed by a Wilcoxon rank sum test with 6-fold Bonferroni correction. Comparisons between group 2-8 were performed by a Wilcoxon rank sum test. The direction of the effect is indicated with +/- signs, where +/- * p<0.01, ++/-- p<0.01, +++/--- p<0.001.

Group	Description	group 2<br>SLA-LSQ 50:50	group 3<br>SLA-LSQ 25:25	group 4<br>SLA-LSQ 10:25	group 5<br>SLA-LSQ 5:2	group 6<br>$Al(OH)_3$ +NaCl	group 7<br>$AS01_B$
1	no adjuvant	<0.001 +++	<0.001 +++	<0.001 +++	0.007 ++	<0.001 +++	<0.001 +++
2	SLA-LSQ 50:50		0.959	0.279	0.001 --	0.328	0.05 +
3	SLA-LSQ 25:25			0.798	0.002 --	0.878	0.195
4	SLA-LSQ 10:25				0.001 --	0.798	0.015 +
5	SLA-LSQ 5:2					0.001 ++	<0.001 +++
6	$Al(OH)_3$+NaCl						0.021+

Supplemental table 6. P-values statistical comparisons ICS CD4 stimulated with FL-HA peptide pool week 10. Comparisons between group 1 and group 2-7 were performed by a Wilcoxon rank sum test with 6-fold Bonferroni correction. Comparisons between group 2-8 were performed by a Wilcoxon rank sum test. The direction of the effect is indicated with +/- signs, where '+' indicates the colum group response > row group response and '-' indicates the colum group response < row group response: +/- * p<0.01, ++/-- p<0.01, +++/--- p<0.001.

Group	Description	group 2<br>SLA-LSQ 50:50	group 3<br>SLA-LSQ 25:25	group 4<br>SLA-LSQ 10:25	group 5<br>SLA-LSQ 5:2	group 6<br>$Al(OH)_3$ +NaCl	group 7<br>$AS01_B$
1	no adjuvant	>0.999	>0.999	0.390	>0.999	>0.999	0.247
2	SLA-LSQ 50:50		>0.999	0.394	0.026-	0.937	0.310
3	SLA-LSQ 25:25	-		0.974	0.132	>0.999	0.310
4	SLA-LSQ 10:25	-			0.009--	0.589	0.065
5	SLA-LSQ 5:2	-				0.026+	0.004++
6	$Al(OH)_3$+NaCl						0.180

Supplemental table 7. P-values statistical comparisons ICS CD4 stimulated with HA stem peptide pool week 10. Comparisons between group 1 and group 2-7 were performed by a Wilcoxon rank sum test with 6-fold Bonferroni correction. Comparisons between group 2-8 were performed by a Wilcoxon rank sum test. The direction of the effect is indicated with +/- signs, where '+' indicates the colum group response > row group response and '-' indicates the colum group response < row group response: +/- * p<0.01, ++/-- p<0.01, +++/--- p<0.001.

Group	Description	group 2<br>SLA-LSQ 50:50	group 3<br>SLA-LSQ 25:25	group 4<br>SLA-LSQ 10:25	group 5<br>SLA-LSQ 5:2	group 6<br>$Al(OH)_3$ +NaCl	group 7<br>$AS01_B$
1	no adjuvant	0.390	>0.999	>0.999	>0.999	0.026+	0.013+
2	SLA-LSQ 50:50		0.738	0.310	0.015-	0.699	0.065
3	SLA-LSQ 25:25	-		0.699	0.041-	0.394	0.026-
4	SLA-LSQ 10:25	-			0.093	0.132	0.002++
5	SLA-LSQ 5:2	-				0.002++	0.002++
6	$Al(OH)_3$+NaCl						0.026

Supplemental table 8. P-values statistical comparisons ICS CD4 stimulated with FL-HA peptide pool week 36. Comparisons between group 1 and group 2-7 were performed by a Wilcoxon rank sum test with 6-fold Bonferroni correction. Comparisons between group 2-8 were performed by a Wilcoxon rank sum test. The direction of the effect is indicated with +/- signs, where '+' indicates the colum group response > row group response and '-' indicates the colum group response < row group response: +/- * p<0.01, ++/-- p<0.01, +++/--- p<0.001.

Group	Description	group 2<br>SLA-LSQ 50:50	group 3<br>SLA-LSQ 25:25	group 4<br>SLA-LSQ 10:25	group 5<br>SLA-LSQ 5:2	group 6<br>$Al(OH)_3$ +NaCl	group 7<br>$AS01_B$
1	no adjuvant	>0.999	>0.999	>0.999	>0.999	>0.999	0.685
2	SLA-LSQ 50:50		0.161	0.798	0.824	0.721	0.054
3	SLA-LSQ 25:25			0.083	0.038 -	0.279	0.336
4	SLA-LSQ 10:25				0.328	0.721	0.014+
5	SLA-LSQ 5:2					0.798	0.004++
6	$Al(OH)_3$+NaCl						0.072

Supplemental table 9. P-values statistical comparisons ICS CD4 stimulated with HA stem peptide pool week 36. Comparisons between group 1 and group 2-7 were performed by a Wilcoxon rank sum test with 6-fold Bonferroni correction. Comparisons between group 2-8 were performed by a Wilcoxon rank sum test. The direction of the effect is indicated with +/- signs, where '+' indicates the colum group response > row group response and '-' indicates the colum group response < row group response: +/- * p<0.01, ++/-- p<0.01, +++/--- p<0.001.

Group	Description	group 2<br>SLA-LSQ 50:50	group 3<br>SLA-LSQ 25:25	group 4<br>SLA-LSQ 10:25	group 5<br>SLA-LSQ 5:2	group 6<br>$Al(OH)_3$ +NaCl	group 7<br>$AS01_B$
1	no adjuvant	>0.999	0.216	>0.999	>0.999	>0.999	0.013+
2	SLA-LSQ 50:50		0.574	0.798	0.005--	0.959	0.040+
3	SLA-LSQ 25:25			0.065	0.007--	0.161	0.232
4	SLA-LSQ 10:25				0.010-	0.959	0.004++
5	SLA-LSQ 5:2					0.005++	0.001+++
6	$Al(OH)_3$+NaCl						0.021+
